# Factors associated with childhood chronic malnutrition during the first 12 months of life in children from a peruvian cohort

**DOI:** 10.17843/rpmesp.2025.421.13662

**Published:** 2025-03-18

**Authors:** Bladimir Morales-Cahuancama, Elena Gonzales-Achuy, Gilmer Solis-Sánchez, Cinthia Quispe-Gala, William Bautista-Olortegui, Gabriela Santos-Antonio, Paúl Hinojosa, Juan Pablo Aparco

**Affiliations:** 1 National Center for Food, Nutrition, and Healthy Living, Instituto Nacional de Salud, Lima, Peru. National Center for Food, Nutrition, and Healthy Living Instituto Nacional de Salud Lima Peru; 2 Academic Program in Nutrition and Dietetics, Faculty of Health Sciences, Universidad Peruana de Ciencias Aplicadas, Lima, Peru. Universidad Peruana de Ciencias Aplicadas Academic Program in Nutrition and Dietetics Faculty of Health Sciences Universidad Peruana de Ciencias Aplicadas Lima Peru

**Keywords:** Undernutrition, Stunting, Infant, Cohort, Maternal Height, Low and Middle Income Countries

## Abstract

**Objective.:**

To determine the factors associated with chronic child malnutrition (CCM) in two Peruvian regions: Huancavelica and Loreto.

**Materials and methods.:**

We analyzed four repeated cross-sectional evaluations nested in a prospective cohort of children treated in primary healthcare facilities. Data was collected on the characteristics of the child, the mother and the household, including anthropometric information, breastfeeding, complementary feeding, and socioeconomic conditions. A generalized linear model was used to calculate the prevalence ratios (PR) for each of the four quarterly evaluations of the cohort (3rd, 6th, 9th and 12th months).

**Results.:**

Factors such as male sex, bottle feeding and the use of polluting fuels were associated with a higher risk of CCM, on the other hand, having six or more prenatal check-ups and being a beneficiary of the JUNTOS program acted as protective factors. Mothers who did not speak Spanish as their native language, who worked or studied, or who had limited participation in family decisions, were also at higher risk of CCM. In addition, we identified unexpected associations, such as food insecurity acting as a protective factor, reflecting possible biases or confounding effects.

**Conclusions.:**

Our findings highlight the importance of addressing inequalities in prenatal care, dietary practices, and mothers’ participation in household decision-making to prevent CCM in vulnerable contexts.

## INTRODUCTION

Chronic child malnutrition (CCM) affects 22% of children under the age of 5 worldwide ^1^. Despite significant improvements, CCM remains a global public health problem ^2^. CCM has consequences for children’s physical growth and neurodevelopment ^3^. Immediate repercussions include a significant increase in infant mortality and greater susceptibility to infections ^4^. In addition, motor, cognitive, socio-emotional, and behavioral deficiencies occur during child neurodevelopment ^5^. CCM is more prevalent in low- and middle-income countries (LMICs), affecting the human capital of the population and plunging LMICs into a vicious cycle of poverty ^6^.

In Peru, the decline in CCM has slowed in recent years ^7^. At the subnational level, regions such as Huancavelica and Loreto have CCM prevalence rates above the national average ^8^. Despite efforts to identify factors associated with CCM (socioeconomic factors and access to health services), most studies are cross-sectional and address populations with wide age ranges (<5 years), or are secondary analyses of surveys that do not collect all the necessary information on factors that may be associated with CCM ^9^^,^^10^.

There are few longitudinal studies conducted in LMICs that assess the effects of household, maternal, and child care variables ^11^^-^^13^, especially in the first year of life, when growth is faster and feeding varies from breast milk to solid foods. In addition, the first infections occur ^14^. Contrary to cross-sectional studies that report CCM occurs at an older age, a study of 32 longitudinal cohorts revealed that the onset of CCM occurs mainly during the first months of life ^15^. This highlights the importance of longitudinal studies in showing that CCM is not only cumulative but also begins to develop early. It also allows for the identification of CCM determinants that vary during the first year of life, providing a more dynamic understanding of the factors that contribute to its development.

Given Peru’s socioeconomic, cultural, and biological diversity, and the complex changes that occur during the first year of life, it is necessary to study the etiology of CCM at the local level in order to implement effective interventions. Therefore, this study aimed to identify factors associated with CCM during the first year of life through quarterly cross-sectional assessments in a prospective cohort in two regions of Peru. This methodological approach takes into account the heterogeneity of feeding characteristics and lifestyles throughout the study period.

**KEY MESSAGES**

Motivation for the study. Chronic child malnutrition (CCM) remains a critical problem, especially in regions such as Huancavelica and Loreto. The specific factors that affect children during their first year of life are not fully understood, in spite of existing programs aimed at this issue.

Main findings. Being male, bottle feeding, and using polluting fuels were identified as factors that increase the risk of CCM, while attending six or more prenatal checkups and receiving support from the JUNTOS program help prevent it. Factors such as maternal language and limited maternal participation also play a role.

Implications for public health. These findings highlight the need for policies that strengthen prenatal care, promote healthy feeding practices, and empower mothers in decision-making.

## MATERIALS AND METHODS

### Study design

Observational study with repeated cross-sectional evaluations, nested in a fixed prospective cohort of Peruvian children aged 3 to 12 months. Four quarterly evaluations were conducted through scheduled home visits since the first month since the evaluation until 15 days after. In each phase, children diagnosed with CCM at the evaluation were excluded, thus allowing the study to focus on potentially healthy children. The study was conducted in two regions: Huancavelica, located in the central highlands at altitudes ranging from 2,661 to 4,274 meters above sea level; and Loreto, located in a jungle plain in the northeast of the country.

The cross-sectional evaluations were carried out independently in order to stratify the observations. This approach allows for the identification of characteristics of children at 3, 6, 9, and 12 months of age and the exploration of determinants associated with the onset of CCM.

### Population and sample

The study population comprised children under one year of age in Huancavelica and Loreto who were receiving care at health facilities (HF) of the Ministry of Health (MINSA). A minimum sample of 660 children was calculated for the two regions, therefore, we enrolled 1373 children to compensate for possible sample attrition (Supplementary Material Figure S1). Additional details on the sampling process and the impact of follow-up losses are available in a previous methodological publication ^16^.

### Enrollment

Initially, birth records were obtained in coordination with MINSA health facilities providing primary care in urban areas, where most births occur, which facilitated enrollment. We included HFs that, according to the local registry of the MINSA Reproductive Health Strategy, registered an average of more than 20 births per month during 2014-2015. Based on this registry, we conducted a baseline census that included all newborns from August 18, 2016, in both regions, extending until October 19 in Loreto and November 23 of the same year in Huancavelica.

Once the initial registry was established, participants were enrolled via home visits during the child’s first month of life, between August and December 2016. Children from multiple pregnancies, with congenital malformations, premature babies, or those born with low birth weight were excluded ^16^.

### Follow-up

Each cross-sectional evaluation of the cohort was carried out through home visits by trained study staff. Losses to follow-up were mainly due to migration or relocation of families, as well as refusal to continue participating in the study ^16^. Interviewers were instructed to make up to three additional attempts to contact the family if they were not found, returning to the home at different times to increase the likelihood of completing the evaluations.

### Variables

The variable of interest was the presence of CCM in each phase of the cohort, determined by Z-scores for height for age less than -2 standard deviations below the median of the standard growth curve for age and sex. Each evaluation provided information on covariates grouped according to: (a) child health and care characteristics, (b) maternal health and knowledge characteristics, and (c) household characteristics.

Child health and care characteristics included aspects self-reported by the mother, such as: (a) breastfeeding information: initiation (within the first hour after birth), exclusive breastfeeding (only breast milk at 3 and 6 months), continued breastfeeding (9 and 12 months), use of bottles for milk or other liquids (due to risk of illness at 6, 9, and 12 months); (b) complementary feeding information: start of food introduction (6th month), minimum frequency of food (9th and 12th months), minimum food diversity (9th and 12th months); (c) growth and development monitoring (CRED), according to the MINSA schedule: number of complete CREDs for age, verified in the health card or reported by the mother (3rd, 6th, 9th and 12th months), complete vaccinations for age, verified in the health card (3rd, 6th, 9th and 12th months); (d) Supplementation: diagnosis of anemia (6th and 12th months), consumption of supplements, reported by the mother (6th, 9th and 12th months), adequate consumption of iron supplements (receipt vs. consumption at 6th, 9th and 12th month); (e) presence of infections: episodes of acute diarrhea (ADA) (3rd, 6th, 9th and 12th month), respiratory infection (ARI) (3rd, 6th, 9th and 12th month). Data were also obtained from primary sources, such as anthropometric and hemoglobin data on the child and information from medical records, such as the child’s birth weight and height (Supplementary Material Table S1).

Mother’s health and knowledge: (a) general characteristics: age (reported by mother), mother with 3 or more children (yes/no), mother tongue, educational level (primary school or less, secondary school and higher), working or studying; (b) pregnancy information: maternal height (anthropometric measurement), pre-pregnancy weight (verified in health card or reported), number of prenatal checkups (PC) (6 or more checkups) and maternal-reported gestational anemia; (c) Postpartum information: participation in demonstration sessions on complementary feeding (at 6, 9, and 12 months), home visits received from the HF (at 6, 9, and 12 months); (d) Other: mother’s participation in important household decisions (at 3, 6, 9, and 12 months); knowledge of nutrition through questions according to the child’s age (Supplementary Material Table S1).

Characteristics of the child’s household: (a) number of household members, (b) having at least one unmet basic need (UBN), and (c) whether the household is a beneficiary of the conditional cash transfer social program (JUNTOS); regarding housing, the following were classified as adequate or inadequate: (d) water source, and (e) fuel for cooking (Supplementary Material Table S1).

### Statistical methods

Children with missing values for the study variables were excluded from the analysis for each quarterly assessment. The distribution of the sample for each variable was characterized using descriptive statistics, employing frequencies and percentages for categorical variables and mean with standard deviation for numerical variables.

The difference in the proportion of CCM according to the variables was evaluated using Pearson’s chi-square test and Fisher’s exact test (according to Cochran’s rule). The numerical variables were also compared between those with and without CCM using Student’s t-test for independent samples and the Mann-Whitney U test (according to the normality of the distribution).

In order to identify factors associated with CCM, we applied a generalized linear binomial family model with Log link and cluster option by region (Huancavelica and Loreto) to correct for variability in the origin of the children; this modeling allowed the calculation of prevalence ratios (PR) of the variables in each quarterly evaluation, then the variables with a p-value less than 0.2 in the crude model were included in the adjusted model, following a statistical adjustment criterion ^17^. Variables that presented collinearity in the crude models and multicollinearity in the adjusted model were excluded when inflation variance scores greater than 10 were found.

Statistical analyses were stratified for each cross-sectional evaluation, using the statistical program STATA version 17.0 (Stata Corporation, USA) with a significance level of 0.05.

In addition, considering that each time point evaluated comprises subsamples of different sizes because they include only children susceptible to the outcome, the power of each model was evaluated independently using the G*Power program version 3.1.9.6 (Universisät Kiel, Germany). In each of the four evaluations, the statistical power (1-b) was 80%.

### Ethical considerations

The study was approved by the Institutional Research Ethics Committee of the National Institute of Health under protocol code: OI-044-15.

## RESULTS

We enrolled 1,373 children in Huancavelica and Loreto. After quality control of the database, some participants were excluded, resulting in 968 surveys included for analysis at 3 months and 513 children at 12 months (Supplementary Material Figure S1).

With regard to the characteristics of the children, we found that the proportion of children with CRED and complete vaccinations decreased as they got older, while ADA and ARI increased. Regarding infant feeding, 39.2% of children aged 6 months had exclusive breastfeeding; bottle use increased considerably in the 9th and 12th months, with almost all children continuing to be breastfed. The prevalence of anemia was high (>49%) in the second and fourth evaluations, while the prevalence of chronic malnutrition was low in all evaluations (Table 1).

**Table 1:** Characteristics of children included in each evaluation in the cohort of children under one year of age. Huancavelica and Loreto, Peru, 2016.

Variables		3 months	6 months	9 months	12 months
		N= 968	N= 814	N= 685	N =513
		n (%)	n (%)	n (%)	n (%)
Region					
	Huancavelica	407 (42.1)	318 (39.1)	286 (41.8)	219 (42.7)
	Loreto	561 (57.9)	496 (60.9)	399 (58.2)	294 (57.3)
Sex					
	Male	483 (49.9)	396 (48.6)	327 (47.7)	229 (44.6)
	Female	485 (50.1)	418 (51.4)	358 (52.3)	284 (55.4)
Child covered by health insurance ^a^					
	No	7 (0.7)	8 (1.0)	6 (0.9)	11 (2.1)
	Yes	961 (99.3)	806 (99.0)	679 (99.1)	502 (97.9)
Children with complete CRED controls ^a^					
	No	286 (29.5)	252 (31.0)	249 (36.4)	240 (46.8)
	Yes	682 (70.5)	562 (69.0)	436 (63.6)	273 (53.2)
Children with complete vaccinations for their age ^a^					
	No	169 (17.5)	425 (52.2)	488 (71.2)	506 (98.6)
	Yes	799 (82.5)	389 (47.8)	197 (28.8)	7 (1.4)
Children with episodes of diarrhea ^a^					
	No	770 (79.5)	601 (73.8)	422 (61.6)	314 (61.2)
	Yes	198 (20.5)	213 (26.2)	263 (38.4)	199 (38.8)
Children with coughing episodes ^a^					
	No	508 (52.5)	387 (47.5)	182 (26.6)	176 (34.3)
	Yes	460 (47.5)	427 (52.5)	503 (73.4)	337 (65.7)
Breastfed within the first hour of birth ^a^					
	No	407 (42.0)	343 (42.1)	288 (42.0)	223 (43.5)
	Yes	561 (58.0)	471 (57.9)	397 (58.0)	290 (56.5)
Exclusive breastfeeding for the first six months of life ^a^					
	No	283 (29.2)	495 (60.8)	405 (59.1)	301 (58.7)
	Yes	685 (70.8)	319 (39.2)	280 (40.9)	212 (41.3)
Bottle feeding ^a^					
	No	866 (89.5)	663 (81.4)	382 (55.8)	298 (58.1)
	Yes	102 (10.5)	151 (18.6)	303 (44.2)	215 (41.9)
Introduction of solid, semi-solid, or soft foods at 6 months ^a^					
	No	--	23 (2.8)	18 (2.6)	14 (2.7)
	Yes	--	791 (97.2)	667 (97.4)	499 (97.3)
Continuous breastfeeding ^a^					
	No	--	--	25 (3.6)	38 (7.4)
	Yes	--	--	660 (96.4)	475 (92.6)
Minimum food diversity ^a^					
	No	--	--	136 (19.9)	55 (10.7)
	Yes	--	--	549 (80.1)	458 (89.3)
Minimum frequency of meals ^a^					
	No	--	--	16 (2.3)	3 (0.6)
	Yes	--	--	669 (97.7)	510 (99.4)
Iron supplement intake ^a^					
	No	--	107 (13.1)	0 (0.0)	0 (0.0)
	Yes	--	707 (86.9)	685 (100)	513 (100)
Daily intake of iron supplements ^a^					
	No	--	--	513 (74.9)	324 (63.2)
	Yes	--	--	172 (25.1)	189 (36.8)
Anemia (<11 mg/dL hemoglobin) ^a^					
	No	--	415 (51.0)	--	240 (46.8)
	Yes	--	399 (49.0)	--	273 (53.2)
Chronic malnutrition ^a^					
	No	928 (95.9)	777 (95.5)	646 (94.3)	491 (95.7)
	Yes	40 (4.1)	37 (4.5)	39 (5.7)	22 (4.3)

N: total number of subjects in the sample, n: number of subjects in the category, %: percentage of subjects in the category

a Last three months prior to the survey.

With regard to the characteristics of the mothers and households (Table 2), most had Spanish as their mother tongue and had completed secondary education. The proportion of mothers who split their time between caring for their children and working or studying increased as follow-up progressed. Coverage of demonstration sessions and home visits in the last three months prior to each assessment was low (<9.4%). Likewise, most mothers participated in key household decisions such as taking the child to the HF and activities related to their feeding. Regarding household characteristics, a considerable proportion did not have food security; the highest proportion of households had a safe water source and adequate fuel for cooking. The complete results of the characteristics of children, mothers, and households are presented in Table S2 of the supplementary material.

**Table 2:** Characteristics of mothers and households included in each assessment of the cohort of children under one year of age in Huancavelica and Loreto, Peru, 2016.

Variables		3 months	6 meses	9 months	12 months
		N= 968	N= 814	N= 685	N =513
		n (%)	n (%)	n (%)	n (%)
	Mother’s age (mean ± SD)	26.1±6.6	26.1±6.7	26.4±6.7	27.5±6.8
	Pre-pregnancy weight of mother (mean ± SD)	57.1±9.4	57.3±9.6	57.9±9.6	58.2±9.8
	Mother’s height (mean ± SD)	151.3±5.0^b^	151.3±5.0	151.6±5.0	151.7±5.0
Mother tongue					
	Other	235 (24.3)	179 (22.0)	168 (24.5)	138 (26.9)
	Spanish	733 (75.7)	635 (78.0)	517 (75.5)	375 (73.1)
Mother’s educational level					
	Primary education or lower	235 (24.3)	200 (24.6)	167 (24.4)	135 (26.3)
	Secondary education	544 (56.2)	453 (55.7)	379 (55.3)	275 (53.6)
	Higher education	189 (19.5)	161 (19.8)	139 (20.3)	103 (20.1)
Mother worked and/or studied ^a^					
	No	622 (64.3)	446 (54.8)	291 (42.5)	211 (41.1)
	Yes	346 (35.7)	368 (45.2)	394 (57.5)	302 (58.9)
Mother with 3 or more children					
	No	786 (81.2)	662 (81.3)	549 (80.1)	414 (80.7)
	Yes	182 (18.8)	152 (18,7)	136 (19.9)	99 (19.3)
Mother with 6 or more prenatal checkups					
	No	167 (17.3)	130 (16.0)	102 (14.9)	67 (13.1)
	Yes	801 (82.7)	684 (84.0)	583 (85.1)	446 (86.9)
Pre-pregnancy anemia					
	No	586 (60.5)	496 (60.9)	416 (60.7)	314 (61.2)
	Yes	382 (39.5)	318 (39.1)	269 (39.3)	199 (38.8)
Participation in demonstrative sessions at HFs ^a^					
	No	--	783 (96.2)	635 (92.7)	465 (90.6)
	Yes	--	31 (3.8)	50 (7.3)	48 (9.4)
Received a home visit from HFs staff ^a^					
	No	--	801 (98.4)	655 (95.6)	484 (94.3)
	Yes	--	13 (1.6)	30 (4.4)	29 (5.7)
Participates in making key decisions at home ^a^					
	No	450 (46.5)	296 (36.4)	224 (32.7)	195 (38.0)
	Yes	518 (53.5)	518 (63.6)	461 (67.3)	318 (62.0)
Household benefiting from the conditional money transfer program (JUNTOS) ^a^					
	No	748 (77.3)	655 (80.5)	529 (77.2)	377 (73.5)
	Yes	220 (22.7)	159 (19.5)	156 (22.8)	136 (26.5)
Food security at home (LACFSS) ^a^					
	No	721 (74.5)	531 (65.2)	380 (55.5)	241 (47.0)
	Yes	247 (25.5)	283 (34.8)	305 (44.5)	272 (53.0)
Household with at least one UBN ^a^					
	No	0 (0.0)	0 (0.0)	0 (0.0)	0 (0.0)
	Yes	968 (100)	814 (100)	685 (100)	513 (100)
Number of household members		6.0 ±2.4	5.9 ±2.4	6.1 ±2.3	5.8 ±2.3
Nutrition knowledge score ^a^		3.3 ±1.0	4.4 ±1.8	3.7 ±1.9	2.4 ±1.1
Water source in the household ^a^					
	Not certain	311 (32.1)	265 (32.6)	206 (30.1)	150 (29.2)
	Certain	657 (67.9)	549 (67.4)	479 (69.9)	363 (70.8)
Main fuel for cooking ^a^					
	Highly polluting	238 (24.6)	211 (25.9)	182 (26.6)	122 (23.8)
	Less polluting	730 (75.4)	603 (74.1)	503 (73.4)	391 (76.2)

CRED: growth and development checkups, HF: health care facility, UBN: unmet basic need, N: total number of subjects in the sample, n: number of subjects in the category, %: percentage of subjects in the category, SD: standard deviation of the variable in the sample, LACFSS: Latin American and Caribbean Food Security Scale.

a During the three months prior to the survey, ^b^ measured in 846 women.

Table 3 presents the bivariate analysis for each cross-sectional assessment. It should be noted that the table shows only the variables with significant differences in at least one of the evaluations. At six months, significant differences were found in the variables of complete CRED (p-value = 0.047), bottle feeding (p-value = 0.026), and child sex (p-value = 0.001). At 9 months, there were significant differences with mothers who worked or studied (p-value = 0.032) and received a demonstration session in the previous months (p-value = 0.005). At 12 months, there were only differences in the household water source variable (p-value = 0.002). The complete results are presented in Table S3 of the supplementary material.

**Table 3:** Factors associated (categorical) with CCM according to bivariate analysis in the four evaluations of the cohort of children under one year of age in Huancavelica and Loreto, Peru, 2016.

Variables		CCM at 3 months		CCM at 6 months		CCM at 9 months		CCM at 12 months	
		n (%)	p-value	n (%)	p-value	n (%)	p-value	n (%)	p-value
Children’s characteristics									
Region									
	Huancavelica	19 (4.7)	0.475 ^a^	16 (5.0)	0.594 ^a^	24 (8.4)	0.010 ^a^	13 (5.9)	0.112 ^a^
	Loreto	21 (3.7)		21 (4.2)		15 (3.8)		9 (3.1)	
Sex									
	Male	24 (5.0)	0.192 ^a^	28 (7.1)	0.001 ^a^	24 (7.3)	0.076 ^a^	11 (4.8)	0.605 ^a^
	Female	16 (3.3)		9 (2.2)		15 (4.2)		11 (3.9)	
Child covered by health insurance									
	No	0 (0.0)	1.000 ^b^	0 (0.0)	1.000 ^b^	0 (0)	1.000 ^b^	1 (9.1)	0.386 ^b^
	Yes	40 (4.2)		37 (4.6)		39 (5.7)		21 (4.2)	
Children with complete CRED checkups^c^									
	No	11 (3.9)	0.772 ^a^	6 (2.4)	0.047 ^a^	12 (4.8)	0.456 ^a^	7 (2.9)	0.150 ^a^
	Yes	29 (4.3)		31 (5.5)		27 (6.2)		15 (5.5)	
Complete vaccinations for the child's age ^c^									
	No	6 (3.6)	0.676 ^a^	20 (4.7)	0.818 ^a^	29 (5.9)	0.658 ^a^	21 (4.2)	0.266 ^b^
	Yes	34 (4.3)		17 (4.4)		10 (5.1)		1 (14.3)	
Children with episodes of diarrhea ^c^									
	No	29 (3.8)	0.259 ^a^	23 (3.8)	0.098 ^a^	24 (5.7)	0.993 ^a^	16 (5.1)	0.257 ^a^
	Yes	11 (5.6)		14 (6.6)		15 (5.7)		6 (3.0)	
Children with coughing episodes ^c^									
	No	17 (3.4)	0.197 ^a^	16 (4.1)	0.592 ^a^	13 (7.1)	0.325 ^a^	5 (2.8)	0.242 ^a^
	Yes	23 (5.0)		21 (4.9)		26 (5.2)		17 (5.0)	
Breastfed within the first hour of birth									
	No	18 (4.4)	0.699 ^a^	13 (3.8)	0.377 ^a^	12 (4.2)	0.142 ^a^	7 (3.1)	0.260 ^a^
	Yes	22 (3.9)		24 (5.1)		27 (6.8)		15 (5.2)	
Exclusive breastfeeding for the first six months of life ^c^									
	No	10 (3.5)	0.548 ^a^	25 (5.1)	0.389 ^a^	16 (4)	0.018 ^a^	10 (3.3)	0.198 ^a^
	Yes	30 (4.4)		12 (3.8)		23 (8.2)		12 (5.7)	
Bottle feeding									
	No	35 (4.0)	0.602 ^b^	25 (3.8)	0.026 ^a^	24 (6.3)	0.455 ^a^	14 (4.7)	0.590 ^a^
	Yes	5 (4.9)		12 (8.0)		15 (5)		8 (3.7)	
Introduction of solid, semi-solid, or soft foods at 6 months									
	No	--	--	0 (0.0)	0.619 ^b^	0 (0)	0.617 ^b^	0 (0.0)	1.000 ^b^
	Yes	--		37 (4.7)		39 (5.9)		22 (4.4)	
Continuous breastfeeding									
	No	--	--	--	--	1 (4)	1.000 ^b^	1 (2.6)	1.000 ^b^
	Yes	--		--		38 (5.8)		21 (4.4)	
Minimum food diversity									
	No	--	--	--	--	6 (4.4)	0.471 ^a^	1 (1.8)	0.495 ^b^
	Yes	--		--		33 (6)		21 (4.6)	
Minimum frequency of meals									
	No	--	--	--	--	0 (0.0)	1.000 ^b^	0 (0.0)	1.000 ^b^
	Yes	--		--		39 (5.8)		22 (4.3)	
Iron supplement intake ^c^									
	No	--	--	4 (3.7)	0.807 ^b^	--	NC	--	NC
	Yes	--		33 (4.7)		39 (5.7)		22 (4.3)	
Anemia (<11 mg/dL hemoglobin)									
	No	--	--	19 (4.6)	0.963 ^a^	--	--	15 (4.8)	0.493 ^a^
	Yes	--		18 (4.5)		--		7 (3.5)	
Characteristics of mothers and households									
Mother tongue									
	Other	11 (4.7)	0.627 ^a^	12 (6.7)	0.116 ^a^	14 (8.3)	0.089 ^a^	9 (6.5)	0.130 ^a^
	Spanish	29 (4.0)		25 (3.9)		25 (4.8)		13 (3.5)	
Mother’s educational level									
	Primary education or lower	13 (5.5)	0.421 ^a^	9 (4.5)	0.587 ^a^	13 (7.8)	0.096 ^a^	8 (5.9)	0.547 ^a^
	Secondary education	19 (3.5)		23 (5.1)		23 (6.1)		10 (3.6)	
	Higher education	8 (4.2)		5 (3.1)		3 (2.2)		4 (3.9)	
Mother worked and/or studied ^c^									
	No	22 (3.5)	0.212 ^a^	22 (4.9)	0.559 ^a^	23 (7.9)	0.032 ^a^	6 (2.8)	0.177 ^a^
	Yes	18 (5.2)		15 (4.1)		16 (4.1)		16 (5.3)	
Mother with 3 or more children									
	No	29 (3.7)	0.150 ^a^	30 (4.5)	0.969 ^a^	28 (5.1)	0.178 ^a^	17 (4.1)	0.592 ^b^
	Yes	11 (6.0)		7 (4.6)		11 (8.1)		5 (5.1)	
Mothers with 6 or more prenatal checkups									
	No	10 (6.0)	0.185 ^a^	9 (6.9)	0.156 ^a^	5 (4.9)	0.708 ^a^	2 (3.0)	0.754 ^b^
	Yes	30 (3.8)		28 (4.1)		34 (5.8)		20 (4.5)	
Pre-pregnancy anemia									
	No	24 (4.1)	0.943 ^a^	25 (5.0)	0.397 ^a^	27 (6.5)	0.263 ^a^	15 (4.8)	0.493 ^a^
	Yes	16 (4.2)		12 (3.8)		12 (4.5)		7 (3.5)	
Participation in demonstration sessions at HF ^c^									
	No	--	--	35 (4.5)	0.647 ^b^	31 (4.9)	0.005 ^b^	21 (4.5)	0.710 ^b^
	Yes	--		2 (6.5)		8 (16)		1 (2.1)	
Received a home visit from HF staff ^c^									
	No	--	--	36 (4.5)	0.456 ^b^	35 (5.3)	0.084 ^b^	20 (4.1)	0.357 ^b^
	Yes	--		1 (7.7)		4 (13.3)		2 (6.9)	
Participates in making key decisions in the home ^c^									
	No	21 (4.7)	0.436 ^a^	18 (6.1)	0.112 ^a^	15 (6.7)	0.430 ^a^	10 (5.1)	0.462 ^a^
	Yes	19 (3.7)		19 (3.7)		24 (5.2)		12 (3.8)	
Household benefiting from the conditional money transfer program (JUNTOS) ^c^									
	No	30 (4.0)	0.726 ^a^	29 (4.4)	0.743 ^a^	27 (5.1)	0.220 ^a^	19 (5.0)	0.162 ^a^
	Yes	10 (4.6)		8 (5.0)		12 (7.7)		3 (2.2)	
Food security at the household (ELCSA) ^c^									
	No	29 (4.0)	0.769 ^a^	23 (4.3)	0.688 ^a^	26 (6.8)	0.148 ^a^	13 (5.4)	0.245 ^a^
	Yes	11 (4.5)		14 (5.0)		13 (4.3)		9 (3.3)	
Household water source ^c^									
	Not certain	12 (3.9)	0.768 ^a^	10 (3.8)	0.463 ^a^	8 (3.9)	0.180 ^a^	0 (0.0)	0.002 ^a^
	Certain	28 (4.3)		27 (4.9)		31 (6.5)		22 (6.1)	
Main fuel for cooking ^c^									
	Highly polluting	13 (5.5)	0.235 ^a^	10 (4.7)	0.875 ^a^	10 (5.5)	0.892 ^a^	9 (7.4)	0.054 ^a^
	Less polluting	27 (3.7)		27 (4.5)		29 (5.8)		13 (3.3)	
Total		40 (4.1)		37 (4.6)		39 (5.7)		22 (4.3)	

a Pearson’s chi-square test, ^b^ Fisher’s exact test, ^c^ during the three months prior to the survey, CCM: chronic child malnutrition. NC: not calculable because it is constant.

In addition, Table 4 shows all quantitative variables analyzed in each cohort evaluation. The variable that showed significant differences on more than one occasion was maternal height (p-value <0.001). Pre-pregnancy maternal weight showed differences in the second and third assessments.

**Table 4:** Factors associated (quantitative) with CCM according to bivariate analysis in the four evaluations of the cohort of children under one year of age in Huancavelica and Loreto, Peru, 2016.

Variables	3 months			6 months			9 months			12 months		
	Without CCM M±SD	With CCM M±SD	p-value	Without CCM M±SD	With CCM M±SD	p-value	Without CCM M±SD	With CCM M±SD	p-value	Without CCM M±SD	With CCM M±SD	p-value
Mother’s age	26.0 ±6.6	26.4 ±7	0.663 ^b^	26.2 ±6.6	24.7 ±7	0.113 ^b^	26.4 ±6.7	27.4 ±7.6	0.429 ^b^	27.5 ±6.8	27.5 ±6,0	0.873 ^b^
Mother’s pre-pregnancy weight	56.8 ±9.4	54.1 ±8.2	0.058 ^b^	57.5 ±9.6	53.0 ±7.3	0.009 ^b^	58.1 ±9.5	53.5 ±9.4	0.005 ^b^	58.3 ±9.8	54.5 ±8.7	0.153 ^b^
Mother’s height	151.3 ±5.1	--	NC ^a^	151.5 ±4.9	148.2 ±4.3	<0.001 ^b^	151.8 ±5.0	148.5 ±4.5	<0.001^b^	151.8 ±5	149.6 ±5.3	0.027 ^b^
Number of members in the household	5.9 ±2.4	6 ±1.9	0.438 ^b^	5.9 ±2.3	6.3 ±2.5	0.357 ^b^	6.1 ±2.3	6.2 ±1.9	0.436 ^b^	5.8 ±2.2	5.8 ±2.6	0.713 ^b^
Nutrition knowledge score	3.3 ±0.9	3.3 ±1	0.980 ^c^	4.4 ±1.8	4.4 ±1.5	0.762 ^b^	3.7 ±1.9	3.2 ±1.7	0.114 ^b^	2.4 ±1.1	2.5 ±1.0	0.613 ^c^

CCM: chronic child malnutrition, M: mean, SD: standard deviation.

a Not calculable because it was registered after 6 months, ^b^ Mann-Whitney U test, ^c^ Student’s t-test for independent samples with equal variances.

Table 5 shows the adjusted PRs for the variables for each quarterly evaluation. At 3 months, the risk factors associated with CCM were episodes of cough (PR: 1.48, 95% CI: 1.09, 2.01, p=0.013), mothers with 3 or more children (PR: 1.95, 95% CI: 1.8, 2.12, p<0.001), and mothers who work or study (PR: 1.43, 95% CI: 1.09, 1.87, p=0.010). The protective factors we identified were maternal pre-pregnancy weight (PR: 0.96; 95% CI: 0.95; 0.97; p<0.001) and food insecurity in the household (PR: 0.77; 95% CI: 0.72; 0.82; p<0.001). At 6 months, the risk factors we identified were bottle feeding (PR: 1.83, 95% CI: 1.11; 3, p=0.017) and mothers whose native language was not Spanish (PR: 2.02, 95% CI: 1.79; 2.27, p<0.001). On the other hand, protective factors included not having completed CRED (PR: 0.39; 95% CI: 0.25; 0.6; p<0.001); not breastfeeding within the first hour after birth (PR: 0.8; 95% CI: 0.73; 0.87; p<0.001); mothers who attended most PCs (PR: 0.44; 95% CI: 0.42; 0.45; p<0.001) and not receiving a demonstrative session (PR: 0.83; 95% CI: 0.78; 0.88; p<0.001). At 9 months, the risk factors we identified were male sex (PR: 1.78; 95% CI: 1.70; 1.86; p<0.001), mothers who did not participate in key household decisions (PR: 1.32; 95% CI: 1.29; 1.35; p<0.001) and food-insecure households (PR: 1.49; 95% CI: 1.20; 1.85; p<0.001). Protective factors included not receiving breast milk in the first hour of life (PR: 0.66; 95% CI: 0.63; 0.68; p<0.001) and not receiving exclusive breastfeeding until six months (PR: 0.49; 95% CI: 0.46; 0.52; p<0.001). Finally, at 12 months, risk factors included mothers whose native language was not Spanish (PR: 1.84; 95% CI: 1.31; 2.58; p<0.001), mothers who work and/or study (PR: 1.77; 95% CI: 1.09; 2.88; p=0.021), not having received a demonstrative session (PR: 2.00; 95% CI: 1.84; 2.17; p<0.001), mothers who do not participate in key household decisions (PR: 1.59. 95% CI: 1.51; 1.67. p<0.001) and use of highly polluting fuel (PR: 2.30. 95% CI: 1.05; 5.02. p=0.037). Protective factors included episodes of diarrhea (PR: 0.61. 95% CI: 0.43; 0.87. p=0.007), anemia (PR: 0.76, 95% CI: 0.68, 0.86, p<0.001), and participation in the JUNTOS conditional cash transfer program (OR: 0.3, 95% CI: 0.14, 0.67, p=0.003). The crude and fully adjusted models are summarized in Tables S3 and S4 of the supplementary material.

**Table 5:** Factors associated with chronic child malnutrition (CCM) according to multivariate analysis in the four evaluations of the cohort of children under one year of age in Huancavelica and Loreto, Peru.

**Children’s characteristics**		3 months ^a^		6 months ^b^		9 months ^c^		12 months ^d^	
		PRa (95%CI)	p-value	PRa (95%CI)	p-value	PRa (95%CI)	p-value	PRa (95%CI)	p-value
Sex									
	Male	--		--		1.78	<0.001	--	
						(1.7; 1.86)			
	Female	--		--		Reference		--	
Children with complete CRED checkups									
	No	--		0.39	<0.001	--		--	
				(0.25; 0.6)					
	Yes	--		Reference		--		--	
Children with episodes of diarrhea									
	No	--		--		--		Reference	
	Yes	--		--		--		0.61	0.007
								(0.43; 0.87)	
Children with coughing episodes									
	No	Reference		--		--		--	
	Yes	1.48	0.013	--		--		--	
		(1.09; 2.01)							
Breastfed within the first hour of birth									
	No	--		0.8	<0.001	0.66	<0.001	--	
				(0.73; 0.87)		(0.63; 0.68)			
	Yes	--		Reference		Reference		--	
Exclusive breastfeeding for the first six months of life									
	No	--		--		0.49	<0.001	--	
						(0.46; 0.52)			
	Yes	--		--		Reference		--	
Bottle feeding									
	No	--		Reference		--		--	
	Yes	--		1.83	0.017	--		--	
				(1.11; 3.0)					
Anemia (<11 mg/dL hemoglobin)									
	No	--		--		--		Reference	
	Yes	--		--		--		0,76	<0.001
								(0.68; 0.86)	
Characteristics of mothers and households									
Mother’s pre-pregnancy weight		0.96	<0.001	--		--		--	
		(0.95; 0.97)							
Mother tongue									
	Other	--		2.02	<0.001	--		1.84	<0.001
				(1.79; 2.27)				(1.31; 2.58)	
	Spanish	--		Reference		--		Reference	
Mother worked and/or studied									
	No	Reference		--		--		Reference	
	Yes	1.43	0.01	--		--		1.77	0.021
		(1.09; 1.87)						(1.09; 2.88)	
Mother with 3 or more children									
	No	Reference		--		--		--	
	Yes	1.95	<0.001	--		--		--	
		(1.8; 2.12)							
Mothers with 6 or more prenatal checkups									
	No	--		Reference		--		--	
	Yes	--		0.44	<0.001	--		--	
				(0.42; 0.45)					
Participated in demonstrative sessions at HF									
	No	--		0.83	<0.001	--		2	<0.001
				(0.78; 0.88)				(1.84; 2.17)	
	Yes	--		Reference		--		Reference	
Participated in making key decisions in the household									
	No	--		--		1.32	<0.001	1.59	<0.001
						(1.29; 1.35)		(1.51; 1.67)	
	Yes	--		--		Reference		Reference	
Household benefiting from the conditional money transfer social program (JUNTOS)									
	No	--		--		--		Reference	
	Yes	--		--		--		0.3	0.003
								(0.14; 0.67)	
Food security in the household (ELCSA)									
	No	0.77	<0.001	--		1.49	<0.001	--	
		(0.72; 0.82)				(1.2; 1.85)			
	Yes	Reference		--		Reference		--	
Main fuel for cooking									
	Highly polluting	--		--		--		2.3	0.037
								(1.05; 5.02)	
	Less polluting	--		--		--		Reference	

PRa: adjusted prevalence ratio, 95% CI: 95% confidence interval, CRED: growth and development checkups, HF: health facility.

a Three-month model, omitting variables: “mother's age” due to multicollinearity and adjusted for “children with complete CRED checkups,” “complete vaccinations for the child’s age,” “children with episodes of diarrhea,” “mother tongue,” and “main fuel for cooking.” Statistical power (1-b) of 0.801.

b Model at six months, omitting variables: “mother's height,” ‘mother's weight,’ and ‘received home visit from HF staff’ due to multicollinearity and adjusted for ‘sex,’ ‘children with episodes of diarrhea,’ ‘children with episodes of cough,’ ‘mother’s educational level,’ ‘participates in key household decisions,’ and ”household water source.” Statistical power (1-b) of 0.800.

c Model at nine months, omitting variables: “mother’s age,” “mother’s height,” “mother’s weight,” “participated in demonstration sessions at HF,” and “received home visit from EESS staff” due to multicollinearity and adjusted for “children with coughing episodes,” ” bottle use,” ‘mother's language,’ ‘mother’s educational level,’ ‘mother worked and/or studied,’ ‘mother with 3 or more children,’ ‘mothers with 6 or more prenatal checkups,’ ‘household beneficiary of the conditional cash transfer program (JUNTOS),’ and ”number of household members.” Statistical power (1-b) of 0.803.

d Model at twelve months, omitting variables: “Children with complete vaccinations for their age” and ‘mother’s height’ due to multicollinearity and adjusted for ‘sex’, ‘exclusive breastfeeding for age or up to six months’, ‘minimum food diversity’, ‘mother’s educational level, ‘pre-gestational anemia’ and ‘household food security (ELCSA)’. Statistical power (1-b) of 0.801.

## DISCUSSION

Our research revealed a variety of factors associated with CCM during the first year of life. Some of these factors manifested their influence in a single cross-sectional analysis, such as male sex, bottle use, and use of highly polluting fuels associated with risk; while the protective factors included mothers having six or more PCs and being beneficiaries of the JUNTOS program. Other factors showed their influence in different evaluations, such as mothers whose native language was not Spanish, mothers who worked or studied during the child’s first year of life, and mothers who did not participate in key household decisions, which acted as risk determinants. In addition, factors with controversial effects were identified regarding services aimed at preventing CCM, such as CREDs, demonstrative sessions, or the presence of diseases such as anemia or diarrhea in the child. A finding that has been little explored in the Peruvian context was the relationship between maternal participation in key household decisions and CCM.

### Factors associated with CCM at 3 months

At 3 months, episodes of coughing were identified as a significant risk, in line with studies showing how ARIs compromise nutritional status by increasing metabolic requirements, reducing food intake, and altering the growth hormone axis ^18^^,^^19^. Having three or more children also represented a risk, possibly due to competition for limited resources and reduced maternal care ^20^^,^^21^. Similarly, mothers who were studying or working showed a negative association, which, in vulnerable contexts, could be related to less time for key practices such as exclusive breastfeeding and direct child care ^22^^,^^23^.

On the other hand, maternal pre-pregnancy weight acted as a protective factor, supporting the importance of good maternal nutritional status for early childhood development ^24^. Finally, the paradoxical association of food insecurity as a protective factor could reflect biases in its measurement or confounding effects, such as the prioritization of resources toward the most vulnerable members of the household ^25^.

### Factors associated with CCM at 6 months

Bottle feeding was identified as a risk factor, consistent with studies highlighting its role as a source of infection, especially in settings with limited access to safe drinking water and inadequate hygiene conditions ^26^^,^^27^. Similarly, the risk associated with mothers whose native language was not Spanish reflects socioeconomic disparities and barriers to access to health and education services, which can negatively affect child nutrition ^28^.

Among protective factors, regularly attending PCs was found to reduce the risk of CCM, reinforcing the importance of maternal monitoring during pregnancy and training in child care practices ^29^. However, seemingly contradictory results emerged regarding full attendance at CRED and demonstrative sessions, which were associated with an increased risk of CCM. One possible explanation is that children at higher risk of stunting were identified early, leading to higher attendance at CRED. However, underlying factors such as socioeconomic vulnerability, difficulties in complying with recommendations, and limited access to resources could limit the effectiveness of these interventions.

### Factors associated with CCM at 9 months

Male sex was associated with a higher risk of CCM, possibly linked to differences in gender-specific care, which is consistent with studies indicating greater neonatal and perinatal vulnerability in males ^30^.

The low participation of mothers in household decisions was also a risk factor, supporting research linking limited maternal autonomy with poorer child health outcomes ^31^^,^^32^. Mothers with greater autonomy tend to participate actively in decisions about feeding and care, which promotes protective practices.

Household food insecurity, another risk factor, reflects the multiple ways in which the food crisis affects child growth. This phenomenon not only compromises food availability, but also generates family stress, maternal malnutrition, and changes in parenting practices, which increase the risk of CCM ^33^.

An unexpected finding was that children without exclusive breastfeeding up to six months of age were less likely to have CCM; in this regard, a previous study in Peru reported that this association would reflect inverse causality, while another international study reported similar findings ^34^.

### Factors associated with CCM at 12 months

Similar to our results at 6 months, children whose mothers’ native language was not Spanish were at higher risk of CCM. This finding, which is rarely reported in articles despite its availability in national surveys, reflects its close association with multidimensional poverty, as it is linked to belonging to indigenous groups and conditions of social exclusion ^28^. In Peru, studies have reported that the prevalence of IDA in indigenous children is twice that of non-indigenous children ^35^, underscoring the relevance of this variable in contexts of structural inequality.

Similarly, children of mothers who worked or studied were at greater risk of CCM, possibly due to competition with time spent on childcare. This result is consistent with studies linking double workloads to negative outcomes in children’s health ^22^.

The lack of participation of mothers in key household decisions reinforces its negative impact on child growth, limiting access to resources and informed decisions for child care ^31^^,^^32^.

The use of highly polluting fuels, such as firewood or coal, was also associated with a higher risk of CCM. These fuels increase respiratory infections and indirectly affect nutritional status, as well as serving as an indicator of household socioeconomic status ^36^.

Among protective factors, the negative association between diarrhea and anemia with CCM may be due to the cross-sectional nature of the analysis and a possible reverse causality bias, where affected children received intensive care that mitigated the impact on their growth or its occurrence was very recent without affecting nutritional status. This finding highlights the need to study the frequency and severity of these conditions in longitudinal studies to understand their relationship with CCM.

On the other hand, belonging to the “JUNTOS” social program was identified as a key protective factor, possibly because it facilitates access to services such as CRED and complete vaccination.

Our results highlight the first year of life as a critical period in which multiple factors interact to influence CCM. This underscores the importance of comprehensively assessing social, economic, and environmental determinants in regions such as Huancavelica and Loreto. Linking child growth to cognitive development and establishing nominal monitoring systems will make it possible to identify risks and prevent their impact. These findings support the design of comprehensive policies that include health services, nutrition programs, childcare support, food security, women’s empowerment, and improvements in household sanitation conditions.

The study was conducted from a longitudinal perspective, although the data analysis was cross-sectional, nested within the Peruvian public health system, without altering the regular activities of the HFs. This contrasts with the controlled settings of other follow-up studies, allowing for the collection of more realistic data from a particularly vulnerable child population. In addition, a comprehensive set of variables at the child, mother, and household levels were assessed quantitatively, prospectively, and systematically, providing a comprehensive approach to the study of CCM.

One of the limitations of this study was that some variables were self-reported, which could introduce biases such as social desirability. In addition, although the knowledge questionnaire was not validated, it was based on questions from previous health and nutrition surveys such as ENDES. Another limitation was that no probability sampling was used to select the sample, which could limit the extrapolation of results; however, the census registration of all children born in the HFs ensures, in part, the representativeness of the children for inferring findings to children who attend MINSA HFs in urban areas of Huancavelica and Loreto. Likewise, the non-random assignment of participants to different exposure groups could generate an uneven distribution of confounding factors. In addition, the cross-sectional analysis is a limitation because it does not allow for exploration of the causality of factors for CCM, nor does it avoid reverse causality bias that could exist. To mitigate this problem in part, we collected detailed information on various confounding factors and controlled for by adjusting the multivariate analysis. However, it should also be noted that this analysis considered adjustment for covariates based solely on statistical criteria, which may have underestimated the impact of variables that could theoretically be linked according to the literature in other contexts different from those studied.

In conclusion, several factors associated with CCM were identified during the first year of life, with variations according to the evaluated stage. At three months, risk factors included respiratory infections, maternal participation in work/educational activities, and history of multiple pregnancies. At six months, bottle feeding and a mother tongue other than Spanish increased the risk, while the protective factors were mothers with six or more PC visits, children with incomplete CRED, and not receiving breast milk within the first hour of life. At nine months, male sex, food insecurity, and mothers’ non-participation in key decisions were identified as risk factors, while lack of breastfeeding in the first hour of life and not receiving exclusive breastfeeding until six months showed a protective effect. Finally, at twelve months, risk factors included the use of polluting fuels, a mother tongue other than Spanish, mothers’ non-participation in key decisions, and mothers’ participation in work/academic activities. Protective factors at this age were the presence of diarrhea or anemia, as well as belonging to the JUNTOS program. The persistence of CCM as a public health problem in vulnerable contexts highlights the need to analyze local determinants in detail in order to design tailored and effective interventions.
