# Supplementary material for: Factors associated with childhood chronic malnutrition during the first 12 months of life in children from a peruvian cohort
Source: Rev Peru Med Exp Salud Publica. 2025 Mar 18;42(1):14–27. doi: 10.17843/rpmesp.2025.421.13662 (PMC12176027; doi:10.17843/rpmesp.2025.421.13662)
Supplement: Supplementary material. — Available in the electronic version of the RPMESP. [file rpmesp-42-01-13662-s001.docx]

**MATERIAL SUPLEMENTARIO**

**Figura S1:** Flujograma de seguimiento de los niños menores de 1 año en las 4 fases de la cohorte en Huancavelica y Loreto, Perú

|  | **Seguimiento de la cohorte**  **(Huancavelica y Loreto)** | | | |  | **Análisis del estudio**  **(Huancavelica y Loreto)** | | | |
| --- | --- | --- | --- | --- | --- | --- | --- | --- | --- |
|  |  |  |  |  | |  |  |  |  |
| Inicio del seguimiento |  |  |  |  | |  |  |  |  |
|  |  |  | Niños evaluados:  1508 |  | |  |  |  |  |
|  | Niños con DC:  135 |  |  |  | |  |  |  |  |
|  |  |  | Niños enrolados:  1373 |  | |  |  |  |  |
|  | Pérdidas en seguimiento: 110 |  |  |  | |  |  |  |  |
|  |  |  | Niños evaluados:  1263 |  | |  | Niños evaluados: 1263 |  |  |
| 3° mes | Niños con DC:  48 |  |  |  | |  |  |  | Encuestas incompletas: 295 |
|  |  |  |  |  | |  |  |  |  |
|  | Pérdidas en seguimiento: 168 |  |  |  | |  | Encuestas analizadas: 968 |  |  |
|  |  |  |  |  | |  |  |  |  |
|  |  |  | Niños evaluados:  1047 |  | |  | Niños evaluados: 1047 |  |  |
| 6° mes | Niños con DC:  41 |  |  |  | |  |  |  | Encuestas incompletas: 233 |
|  |  |  |  |  | |  |  |  |  |
|  | Pérdidas en seguimiento: 94 |  |  |  | |  | Encuestas analizadas: 814 |  |  |
|  |  |  |  |  | |  |  |  |  |
|  |  |  | Niños evaluados:  912 |  | |  | Niños evaluados:  912 |  |  |
| 9° mes | Niños con DC:  50 |  |  |  | |  |  |  | Encuestas incompletas: 227 |
|  |  |  |  |  | |  |  |  |  |
|  | Pérdidas en seguimiento: 77 |  |  |  | |  | Encuestas analizadas: 685 |  |  |
|  |  |  |  |  | |  |  |  |  |
|  |  |  | Niños evaluados:  785 |  | |  | Niños evaluados:  785 |  |  |
| 12° mes |  |  |  |  | |  |  |  | Encuestas incompletas: 272 |
|  |  |  |  |  | |  |  |  |  |
|  |  |  |  |  | |  | Encuestas analizadas: 513 |  |  |
|  |  |  |  |  | |  |  |  |  |

**Tabla S1: Operacionalización de las variables**

| **Variable** | **Definición** | **Categorías** | **Unidades de medida** |
| --- | --- | --- | --- |
| **DATOS DEL NIÑO(A)** |  |  |  |
| **Sexo** | Sexo del niño (a) | Masculino | Niño |
|  |  | Femenino | Niña |
| **Edad del niño/a** | Edad en meses | Meses cumplidos | Meses |
| **Peso al nacer** | Peso registrado al nacimiento | Peso | Gramos |
| **Desnutrición crónica** | Medición de la talla para la edad del niño | Desnutrido | T/E menor de -2 DE |
|  |  | Normal | Mayor/ igual a -2DE |
| **Inicio temprano de lactancia materna** | Niños que fueron amamantados dentro del plazo de una hora de su nacimiento | Sí | Inicio temprano de lactancia materna |
|  |  | No | Inicio tardío de lactancia materna |
| **Lactancia materna exclusiva** **(LME)** | Lactantes de 0 a 5 meses de edad alimentados exclusivamente con leche materna | Sí | LME durante los primeros 6 meses de vida del niño |
|  |  | No | LME < 6 meses |
| **Lactancia materna continua** | Niños de 6 -12 meses que fueron amamantados el día anterior | Sí | LME continuada hasta el año de edad |
|  |  | No | LME no continuada |
| **Morbilidad por EDA** | Presencia de episodios de deposiciones liquidas (diarrea) en las ultimas 2 semanas previas a la entrevista | Sí | Presencia de diarrea en las últimas 2 semanas |
|  |  | No |  |
| **Morbilidad por IRA** | Presencia de fiebre o tos en las ultimas 2 semanas previas a la entrevista | Sí | Presencia de fiebre y tos en las últimas 2 semanas |
|  |  | No |  |
| **Seguro de salud** | Niño afiliado a un seguro de salud del niño (seguro integral de salud (SIS), privado, Essalud, otros) | Sí | Niño cuenta con algun seguro de salud |
|  |  | No |  |
| **Controles de crecimiento y desarrollo (CRED)** | Niños que contaban con sus controles CRED completos para la edad | Sí | Niños con controles CRED completos de acuerdo a la edad (minimo 1 control CRED por mes) |
|  |  | No |  |
| **Vacunas** | Niños con vacunas completas para la edad según esquema de vacunación MINSA | Sí | Niños con vacunas cokmpletas de acuerdo a la edad y según el esquema de vacunación MINSA |
|  |  | No |  |
| **Anemia** | Medido a partir de Hemoglobina en sangre | Si | Menor de 11mg/dL |
|  |  | No |  |
| **Introducción de alimentos sólidos, semisólidos o suaves** | Lactantes de 6 a 8 meses de edad que recibieron alimentos sólidos, semisólidos o suaves durante el día anterior, | Sí | Lactantes de 6–8 meses de edad que reciben alimentos sólidos, semisólidos o suaves |
|  |  | No |  |
| **Diversidad mínima de alimentos** | Niños de 6 a 12 meses de edad que recibieron alimentos de ≥ 4 grupos alimentarios durante el día anterior | Sí | Niños de 6 a 12 meses de edad que reciben alimentos de 4 o más grupos de alimentos |
|  |  | No |  |
| **Frecuencia mínima de comidas** | Niños de 6 a 12 meses de edad que recibieron alimentos sólidos, semisólidos o suaves (o leche*****) el número mínimo o más de veces el día anterior, | Sí | Niños de 6 a 12 meses de edad que recibieron que reciben sólidos, semisólidos o suaves (o leche*****) el número mínimo o más |
|  |  | No |  |
| **Dieta mínima aceptable** | Niños amamantados de 6 a 12 meses de edad que tuvieron por lo menos la diversidad alimentaria mínima y la frecuencia mínima de comidas durante el día anterior, | Sí | Niños de 6 a 12 meses de edad que reciben una dieta mínima aceptable (aparte de la leche materna). |
|  |  | No |  |
| **Alimentación con biberón** | Niños de 0 a 12 meses de edad que fueron alimentados con biberón durante el día anterior, | Sí | Niños de 0 a 12 meses de edad que fueron alimentados con biberón |
|  |  | No |  |
| **Consumo de suplemento de hierro** | Niños que consumieron suplemento de hierro desde los 4 meses de edad (jarabe, gotas, chispitas) | Sí | Consumo de suplemento de hierro |
|  |  | No |  |
| **Cuidador principal del niño/a** | Cuidador principal del niño/a | Madre; abuela; padre; hermano; otro | Persona a cargo del niño la mayor parte del día y del tiempo |
| **DATOS DE LA MADRE** |  |  |  |
| **Edad** | Edad | Años cumplidos | Número de años |
| **Peso materno pregestacional** | Peso antes del embarazo | Medido en Kilos | Peso pregestacional |
| **Talla** | Talla | Medido en cms | Estatura de la madre |
| **Lengua materna** | Referido a la lengua que utiliza para comunicarse | Castellano | Se comunica en castellano |
|  |  | Otro | Se comunica en otro tipo de lengua: quechua, aymara, ashaninka, etc.) |
| **Escolaridad de la madre** | Nivel educativo materno | Menos de primaria | Nivel de instrucción alcanzado por la madre |
|  |  | Secundaria completa |  |
|  |  | Superior |  |
| **Número de hijos de la madre** | Madre con 3 hijos o más | Si | Madre con tres hijos o más que vivan con ella |
|  |  | No |  |
| **Con pareja** | Referido a si vive con pareja (conviviente, casada) | Si | Si cuenta con pareja |
|  |  | No |  |
| **Control prenatal (CPN) adecuado** | Número de controles adecuados según mes de embarazo | Sí | 6 ó más CPN |
|  |  | No |  |
| **Anemia** | Diagnóstico de anemia durante el embarazo reportada por la madre | Si | Dato autoreportado por la madre |
|  |  | No |  |
| **Madre trabajó y/o estudió** | Madre estuvo trabajando y/o estudiando durante los 12 meses de edad del niño (a) | Si | Trabajó y/o estudió |
|  |  | No |  |
| **Participa en decisiones importantes en el hogar** | Referido a si la madre decide por ejemplo en el cuidado del niño, alimentación, atención de salud, compra de equipos para el hogar, etc.) | Si | Participación d ela madre en decisiones del hogar |
|  |  | No |  |
| **Recibió sesion demostrativa** | Asistió a sesión demostrativa en el establecimiento de salud para hacer preparaciones saludables y ricos en hierro | Si | Información autoreportada |
|  |  | No |  |
| **Recibió visita domicilaria** | Visita recibida por personal de salud del establecimiento de salud de su jurisdicción | Si | Información autoreportada |
|  |  | No |  |
| **DATOS DEL HOGAR** |  |  |  |
| **Miembros del hogar** | Número de miembros del hogar | Número de personas | Número de personas que habitan en el hogar y comen de la misma olla |
| **Beneficiario de algún programa de asistencia social** | Si la entrevistada se beneficia de algún programa social | Vaso de leche | Beneficiario de algun Programas de apoyo social |
|  |  | Comedor popular |  |
|  |  | Otra asociación o grupo de madres |  |
|  |  | Asociación de regantes |  |
|  |  | Cooperativa de crédito |  |
|  |  | JUNTOS |  |
|  |  | Otros |  |
| **Beneficiario del Programa JUNTOS** | El hogar recibe apoyo del Programa Social de transferencia condicionada - JUNTOS | Si | Recibe apoyo del programa JUNTOS, autoreportado por la madre |
|  |  | No |  |
| **Eliminación de excretas** | Tipo de servicio higiénico que existe en el hogar | Letrina | Observación del tipo de servicio higiénico en el hogar |
|  |  | Conectado a red pública |  |
|  |  | Otros |  |
| **Material del piso** | Material del piso | Tierra | Observación del principal material del piso de la vivienda |
|  |  | Madera |  |
|  |  | cemento |  |
|  |  | otro |  |
| **Fuente de agua** | El hogar cuenta con agua segura | Si | Observación de la conexión de agua (entubada, caño, rio, etc.) |
|  |  | No |  |
| **Fuente de agua** | El hogar cuenta con fuente de agua adecuada | Adecuado | Observación de la fuente adecuada del agua (conexión intradomicialiaria, entubada, caño) |
|  |  | Inadecuado |  |
| **Necesidades básicas Insatisfechas (NBI)** | Hogares con por lo menos 1 NBI | No pobre | Sin NBI |
|  |  | Pobre | Con por lo menos 1 NBI |
| **Seguridad alimentaria** | Seguridad alimentaria (Escala ELCSA) | Seguridad alimentaria | Ninguna respuesta como 1 |
|  |  | Inseguridad alimentaria leve | De 1 a 3 IA leve |
|  |  | Inseguridad alimentaria moderada | De 4 a 6 IA moderada |
|  |  | Inseguridad alimentaria grave | De 7 a 8 IA grave |

**Tabla S2. Características de la muestra de niños enrolados de los niños menores de 1 año en las 4 fases de la cohorte en Huancavelica y Loreto, Perú**

| **Variables** | **Fases de seguimiento** | | | |
| --- | --- | --- | --- | --- |
|  | **3 meses** | **6 meses** | **9 meses** | **12 meses** |
|  | **N=1095 n (%)** | **N=953 n (%)** | **N=835 n (%)** | **N=736 n (%)** |
| **CARACTERÍSTICAS DE LOS NIÑOS** |  |  |  |  |
| Región |  |  |  |  |
| Huancavelica | 473 (43,2) | 415 (43,5) | 353 (42,3) | 302 (41,0) |
| Loreto | 622 (56,8) | 538 (56,5) | 482 (57,7) | 434 (59,0) |
| Sexo |  |  |  |  |
| Hombre | 540 (49,3) | 465 (48,8) | 402 (48,1) | 346 (47,0) |
| Mujer | 555 (50,7) | 488 (51,2) | 433 (51,9) | 390 (53,0) |
| Niño afiliado a un Seguro de salud |  |  |  |  |
| No | 13 (1,2) | 9 (0,9) | 10 (1,2) | 17 (2,3) |
| Si | 1082 (98,8) | 944 (99,1) | 825 (98,8) | 718 (97,6) |
| Datos ausentes | 0 (0,0) | 0 (0,0) | 0 (0,0) | 1 (0,1) |
| Niños con Controles CRED completos* |  |  |  |  |
| No | 298 (27,2) | 290 (30,4) | 317 (38,0) | 375 (51,0) |
| Si | 708 (64,7) | 663 (69,6) | 518 (62,0) | 361 (49,0) |
| Datos ausentes | 89 (8,1) | 0 (0,0) | 0 (0,0) | 0 (0,0) |
| Vacunas completas para la edad del niño(a)* |  |  |  |  |
| No | 172 (15,7) | 443 (46,5) | 599 (71,8) | 728 (98,9) |
| Si | 834 (76,2) | 409 (42,9) | 234 (28,0) | 8 (1,1) |
| Datos ausentes | 89 (8,1) | 101 (10,6) | 2 (0,2) | 0 (0,0) |
| Niños con episodios de diarrea* |  |  |  |  |
| No | 877 (80,1) | 706 (74,1) | 503 (60,2) | 432 (58,7) |
| Si | 218 (19,9) | 247 (25,9) | 332 (39,8) | 304 (41,3) |
| Episodios de tos* |  |  |  |  |
| No | 569 (52,0) | 459 (48,2) | 221 (26,5) | 250 (34) |
| Si | 526 (48,0) | 491 (51,5) | 614 (73,5) | 486 (66) |
| Datos ausentes | 0 (0,0) | 3 (0,3) | 0 (0,0) | 0 (0,0) |
| Amamantados en la primera hora de nacido |  |  |  |  |
| No | 454 (41,5) | 392 (41,1) | 352 (42,2) | 319 (43,3) |
| Si | 638 (58,3) | 561 (58.9) | 483 (57,8) | 417 (56,7) |
| Datos ausentes | 3 (0,3) | 0 (0,0) | 0 (0,0 | 0 (0,0) |
| Lactancia materna exclusiva para la edad o hasta el sexto mes* |  |  |  |  |
| No | 304 (27,8) | 563 (59,1) | 493 (59,0) | 439 (59,7) |
| Si | 788 (72,0) | 390 (40,9) | 342 (41,0) | 297 (40,3) |
| Datos ausentes | 3 (0,2) | 0 (0,0) | 0 (0,0) | 0 (0,0) |
| Uso de biberón |  |  |  |  |
| No | 981 (89,6) | 784 (82,3) | 464 (55,6) | 428 (58,2) |
| Si | 114 (10,4) | 169 (17,7) | 371 (44,4) | 308 (41,8) |
| Introducción de alimentos sólidos, semi-sólidos, o suaves a los 6 meses |  |  |  |  |
| No | ---- | 25 (2,6) | 22 (2,6) | 20 (2,7) |
| Si | ---- | 928 (97,4) | 813 (97,4) | 716 (97,3) |
| Lactancia materna continua |  |  |  |  |
| No | ---- | ---- | 34 (4,1) | 55 (7,5) |
| Si | ---- | ---- | 801 (95,9) | 681 (92,5) |
| Diversidad mínima de alimentos |  |  |  |  |
| No | ---- | ---- | 160 (19,2) | 71 (9,6) |
| Si | ---- | ---- | 662 (79,3) | 652 (88,6) |
| Datos ausentes | ---- | ---- | 13 (1,6) | 13 (1,8) |
| Frecuencia mínima de comidas |  |  |  |  |
| No | ---- | ---- | 18 (2,2) | 4 (0,5) |
| Si | ---- | ---- | 804 (96,3) | 719 (97,7) |
| Datos ausentes | ---- | ---- | 13 (1,6) | 13 (1,8) |
| Consumo suplementos de hierro* |  |  |  |  |
| No | ---- | 133 (14,0) | 8 (1,0) | 84 (11,4) |
| Si | ---- | 820 (86,0) | 827 (99,0) | 652 (88,6) |
| Consumo diario de suplementos de hierro* |  |  |  |  |
| No | ---- | ---- | 614 (73,5) | 415 (56,4) |
| Si | ---- | ---- | 213 (25,5) | 237 (32,2) |
| Datos ausentes | ---- | ---- | 8 (1,0) | 84 (11,4) |
| Anemia (<11mg/dL Hemoglobina) |  |  |  |  |
| No | ---- | 478 (50,2) | ---- | 331 (45,0) |
| Si | ---- | 437 (45,9) | ---- | 405 (55,0) |
| Datos ausentes | ---- | 38 (4) | ---- | 0 (0,0) |
| Desnutrición crónica |  |  |  |  |
| Normal | 1047 (95,6) | 912 (95,7) | 785 (94,0) | 702 (95,4) |
| Desnutrido | 48 (4,4) | 41 (4,3) | 50 (6,0) | 34 (4,6) |
| **CARACTERÍSTICAS DE LAS MADRES Y DEL HOGAR** |  |  |  |  |
| Edad de la madre (X ± DE) | 25,9 ±6,7 | 26.1 ±6.7 | 26.4 ±6.7 | 27.2 ±6.7 |
| Peso de la madre pregestacional (X ± DE) | 56,8 ±9,4 | 57.2 ±9.5 | 57.4 ±9.7 | 57.9 ±9.7 |
| Talla de la madre (X ± DE) | 151,3 ±5,0^a^ | 151.3 ±5.0 | 151.4 ±5.0 | 151.6 ±5.0 |
| Lengua materna |  |  |  |  |
| Otra | 278 (25,4) | 240 (25.2) | 202 (24.2) | 175 (23.8) |
| Castellano | 817 (74,6) | 713 (74.8) | 633 (75.8) | 561 (76.2) |
| Nivel educativo materno alcanzado |  |  |  |  |
| Menos de primaria | 279 (25,5) | 242 (25.4) | 212 (25.4) | 183 (24.9) |
| Secundaria | 610 (55,7) | 532 (55.8) | 459 (55) | 401 (54.5) |
| Superior | 206 (18,8) | 179 (18.8) | 164 (19.6) | 152 (20.7) |
| Madre trabajó y/o estudió* |  |  |  |  |
| No | 690 (63) | 519 (54.5) | 373 (44.7) | 317 (43.1) |
| Si | 405 (37) | 434 (45.5) | 462 (55.3) | 419 (56.9) |
| Madre con 3 hijos o más |  |  |  |  |
| No | 888 (81,1) | 773 (81.1) | 672 (80.5) | 588 (79.9) |
| Si | 207 (18,9) | 180 (18.9) | 163 (19.5) | 148 (20.1) |
| Madres con 6 o más asistencia a controles prenatales |  |  |  |  |
| No | 191 (17,4) | 157 (16.5) | 131 (15.7) | 110 (15) |
| Si | 894 (81,6) | 791 (83) | 699 (83.7) | 621 (84.4) |
| Sin información | 10 (0,9) | 5 (0.5) | 5 (0.6) | 5 (0.7) |
| Anemia pregestacional |  |  |  |  |
| No | 676 (61,7) | 590 (61.9) | 61.92 (61.9) | 61.28 (61.3) |
| Si | 419 (38,3) | 363 (38.1) | 38.08 (38.1) | 38.72 (38.7) |
| Recibió sesión alimentaria demostrativa en EESS* |  |  |  |  |
| No | ----- | 910 (95.5) | 261 (31.3) | 254 (34.5) |
| Si | ----- | 43 (4.5) | 574 (68.7) | 482 (65.5) |
| Recibió visita domiciliaria del personal del EESS* |  |  |  |  |
| No | ----- | 926 (97.2) | 656 (78.6) | 569 (77.3) |
| Si | ----- | 27 (2.8) | 179 (21.4) | 167 (22.7) |
| Participa en tomar decisiones clave en el hogar* |  |  |  |  |
| No | 498 (45,5) | 329 (34.5) | 780 (93.4) | 679 (92.3) |
| Si | 597 (54,5) | 624 (65.5) | 55 (6.6) | 57 (7.7) |
| Hogar beneficiario del programa social de transferencias condicionadas (JUNTOS)* |  |  |  |  |
| No | 846 (77,3) | 757 (79.4) | 799 (95.7) | 701 (95.2) |
| Si | 249 (22,7) | 196 (20.6) | 36 (4.3) | 35 (4.8) |
| Hogar con seguridad alimentaria (ELCSA)* |  |  |  |  |
| No | 821 (75,0) | 628 (65.9) | 470 (56.3) | 358 (48.6) |
| Si | 274 (25,0) | 324 (34) | 354 (42.4) | 365 (49.6) |
| Sin información | 0 (0,0) | 1 (0.1) | 11 (1.3) | 13 (1.8) |
| Hogar con al menos una NBI* |  |  |  |  |
| No | 0 (0,0) | 0 (0,0) | 0 (0,0) | 0 (0,0) |
| Si | 1095 (100) | 953 (100) | 835 (100) | 736 (100) |
| Número de miembros en el hogar | 6,0 ±2,4 | 5.9 ±2.3 | 5.9 ±2.3 | 5.6 ±2.3 |
| Puntaje de conocimientos sobre nutrición* | 3,3 ±1,0 | 4.4 ±1.8 | 3.6 ±1.9 | 2.4 ±1.1 |
| Fuente de agua del hogar* |  |  |  |  |
| No segura | 347 (31,7) | 295 (31) | 260 (31.1) | 233 (31.7) |
| Segura | 748 (68,3) | 658 (69.1) | 575 (68.9) | 503 (68.3) |
| Combustible principal para cocinar* |  |  |  |  |
| Muy contaminantes | 286 (26,1) | 242 (25.4) | 221 (26.5) | 177 (24.1) |
| Menos contaminantes | 809 (73,9) | 711 (74.6) | 614 (73.5) | 559 (76) |
| N: Total de sujetos en la muestra, n: Cantidad de sujetos en la categoría, %: Porcentaje de sujetos en la categoría | | | | |

* Durante los tres meses previos a la encuesta, ^a^ Calculado en 953 personas.

**Tabla S3. Modelos crudos para la determinación de factores asociados a la presencia de DCI según análisis multivariado en las 4 fases de la cohorte de niños menores de 1 año en Huancavelica y Loreto, Perú**

|  | **03 meses^a^** | |  | **06 meses^b^** | |  | **09 meses^c^** | |  | **12 meses^d^** | |
| --- | --- | --- | --- | --- | --- | --- | --- | --- | --- | --- | --- |
|  | **RP cruda (IC 95%)** | **Valor P** |  | **RP cruda (IC 95%)** | **Valor P** |  | **RP cruda (IC 95%)** | **Valor P** |  | **RP cruda (IC 95%)** | **Valor P** |
| **CARACTERÍSTICAS DE LOS NIÑOS** |  |  |  |  |  |  |  |  |  |  |  |
| Sexo |  |  |  |  |  |  |  |  |  |  |  |
| Hombre | 1.51 (0.68; 3.35) | 0.316 |  | 3.28 (0.9; 11.99) | 0.072 |  | 1.75 (1.54; 2) | <0.001 |  | 1.24 (1.07; 1.44) | 0.004 |
| Mujer | Referencia | |  | Referencia | |  | Referencia | |  | Referencia | |
| Niño afiliado a un Seguro de salud |  |  |  |  |  |  |  |  |  |  |  |
| No | ------------- | |  | ------------- | |  | ------------- | |  | 2.17 (0.31; 15.02) | 0.431 |
| Si | ------------- | |  | ------------- | |  | ------------- | |  | Referencia | |
| Niños con Controles CRED completos* |  |  |  |  |  |  |  |  |  |  |  |
| No | 0.90 (0.82; 1.00) | 0.056 |  | 0.43 (0.40; 0.46) | <0.001 |  | 0.78 (0.48; 1.26) | 0.311 |  | 0.53 (0.14; 1.96) | 0.342 |
| Si | Referencia | |  | Referencia | |  | Referencia | |  | Referencia | |
| Vacunas completas para la edad del niño(a)* |  |  |  |  |  |  |  |  |  |  |  |
| No | 1.2 (0.97; 1.48) | 0.094 |  | 1.08 (0.72; 1.60) | 0.715 |  | 1.17 (0.49; 2.77) | 0.720 |  | 0.29 (0.16; 0.53) | <0.001 |
| Si | Referencia | |  | Referencia | |  | Referencia | |  | Referencia | |
| Niños con episodios de diarrea* |  |  |  |  |  |  |  |  |  |  |  |
| No | Referencia | |  | Referencia | |  | Referencia | |  | Referencia | |
| Si | 1.48 (0.9; 2.41) | 0.122 |  | 1.72 (0.81; 3.62) | 0.155 |  | 1 (0.8; 1.25) | 0.980 |  | 0.59 (0.53; 0.67) | <0.001 |
| Episodios de tos* |  |  |  |  |  |  |  |  |  |  |  |
| No | Referencia | |  | Referencia | |  | Referencia | |  | Referencia | |
| Si | 1.49 (1.09; 2.05) | 0.013 |  | 1.19 (1.09; 1.29) | <0.001 |  | 0.72 (0.48; 1.1) | 0.127 |  | 1.78 (0.45; 6.97) | 0.411 |
| Amamantados en la primera hora de nacido |  |  |  |  |  |  |  |  |  |  |  |
| No | 1.13 (0.82; 1.56) | 0.466 |  | 0.74 (0.66; 0.83) | <0.001 |  | 0.61 (0.58; 0.65) | <0.001 |  | 0.61 (0.21; 1.73) | 0.349 |
| Si | Referencia | |  | Referencia | |  | Referencia | |  | Referencia | |
| Lactancia materna exclusiva para la edad o hasta el sexto mes* |  |  |  |  |  |  |  |  |  |  |  |
| No | 0.81 (0.5; 1.3) | 0.379 |  | 1.34 (0.39; 4.57) | 0.638 |  | 0.48 (0.33; 0.71) | <0.001 |  | 0.59 (0.42; 0.83) | 0.002 |
| Si | Referencia | |  | Referencia | |  | Referencia | |  | Referencia | |
| Uso de biberón |  |  |  |  |  |  |  |  |  |  |  |
| No | Referencia | |  | Referencia | |  | Referencia | |  | Referencia | |
| Si | 1.21 (0.4; 3.65) | 0.731 |  | 2.11 (1.26; 3.51) | 0.004 |  | 0.79 (0.57; 1.1) | 0.156 |  | 0.79 (0.23; 2.7) | 0.709 |
| Lactancia materna continua |  |  |  |  |  |  |  |  |  |  |  |
| No | ------------- | |  | ------------- | |  | 0.69 (0.23; 2.09) | 0.517 |  | 0.6 (0.16; 2.16) | 0.430 |
| Si | ------------- | |  | ------------- | |  | Referencia | |  | Referencia | |
| Diversidad mínima de alimentos |  |  |  |  |  |  |  |  |  |  |  |
| No | ------------- | |  | ------------- | |  | 0.73 (0.29; 1.86) | 0.515 |  | 0.4 (0.14; 1.11) | 0.079 |
| Si | ------------- | |  | ------------- | |  | Referencia | |  | Referencia | |
| Consumo suplementos de hierro* |  |  |  |  |  |  |  |  |  |  |  |
| No | ------------- | |  | Referencia | |  | ------------- | |  | ------------- | |
| Si | ------------- | |  | 1.25 (0.49; 3.19) | 0.643 |  | ------------- | |  | ------------- | |
| Anemia (<11mg/dL Hemoglobina) |  |  |  |  |  |  |  |  |  |  |  |
| No | ------------- | |  | Referencia | |  | ------------- | |  | Referencia | |
| Si | ------------- | |  | 0.99 (0.57; 1.7) | 0.958 |  | ------------- | |  | 0.73 (0.62; 0.87) | <0.001 |
| **CARACTERÍSTICAS DE LAS MADRES Y DEL HOGAR** |  |  |  |  |  |  |  |  |  |  |  |
| Edad de la madre | 1.01 (0.99; 1.03) | 0.301 |  | 0.97 (0.86; 1.09) | 0.581 |  | 1.02 (1.01; 1.04) | 0.002 |  | 1 (0.91; 1.09) | 0.999 |
| Peso de la madre pregestacional | 0.97 (0.96; 0.98) | <0.001 |  | 0.95 (0.93; 0.97) | <0.001 |  | 0.95 (0.93; 0.96) | <0.001 |  | 0.96 (0.88; 1.04) | 0.288 |
| Talla de la madre |  |  |  | 0.88 (0.83; 0.93) | <0.001 |  | 0.88 (0.85; 0.91) | <0.001 |  | 0.92 (0.92; 0.92) | <0.001 |
| Lengua materna |  |  |  |  |  |  |  |  |  |  |  |
| Otra | 1.18 (1.17; 1.19) | <0.001 |  | 1.7 (1.17; 2.47) | 0.005 |  | 1.72 (0.91; 3.28) | 0.097 |  | 1.88 (1.37; 2.59) | <0.001 |
| Castellano | Referencia | |  | Referencia | |  | Referencia | |  | Referencia | |
| Nivel educativo materno alcanzado |  |  |  |  |  |  |  |  |  |  |  |
| Menos de primaria | 1.31 (0.84; 2.02) | 0.229 |  | 1.45 (0.46; 4.52) | 0.523 |  | 3.61 (0.47; 27.5) | 0.216 |  | 1.53 (1.43; 1.63) | <0.001 |
| Secundaria | 0.83 (0.25; 2.71) | 0.751 |  | 1.63 (1.33; 2.01) | <0.001 |  | 2.81 (0.6; 13.15) | 0.189 |  | 0.94 (0.5; 1.76) | 0.837 |
| Superior | Referencia | |  | Referencia | |  | Referencia | |  | Referencia | |
| Madre trabajó y/o estudió * |  |  |  |  |  |  |  |  |  |  |  |
| No | Referencia | |  | Referencia | |  | Referencia | |  | Referencia | |
| Si | 1.47 (1.07; 2.02) | 0.017 |  | 0.83 (0.17; 4.1) | 0.815 |  | 0.51 (0.21; 1.26) | 0.144 |  | 1.86 (1.03; 3.38) | 0.041 |
| Madre con 3 hijos o más |  |  |  |  |  |  |  |  |  |  |  |
| No | Referencia | |  | Referencia | |  | Referencia | |  | Referencia | |
| Si | 1.64 (1.5; 1.79) | <0.001 |  | 1.02 (0.23; 4.56) | 0.983 |  | 1.59 (1.23; 2.04) | <0.001 |  | 1.23 (0.41; 3.71) | 0.713 |
| Madres con 6 o más asistencia a controles prenatales |  |  |  |  |  |  |  |  |  |  |  |
| No | 1.6 (0.37; 6.96) | 0.532 |  | 1.69 (1.49; 1.92) | <0.001 |  | 0.84 (0.67; 1.06) | 0.136 |  | 0.67 (0.1; 4.33) | 0.670 |
| Si | Referencia | |  | Referencia | |  | Referencia | |  | Referencia | |
| Anemia pregestacional |  |  |  |  |  |  |  |  |  |  |  |
| No | Referencia | |  | Referencia | |  | Referencia | |  | Referencia | |
| Si | 1.02 (0.28; 3.72) | 0.973 |  | 0.75 (0.31; 1.79) | 0.514 |  | 0.69 (0.33; 1.43) | 0.317 |  | 0.74 (0.56; 0.97) | 0.028 |
| Recibió sesión alimentaria demostrativa en EESS* |  |  |  |  |  |  |  |  |  |  |  |
| No | ------------- | |  | 0.69 (0.47; 1.03) | 0.068 |  | 0.31 (0.21; 0.45) | <0.001 |  | 2.17 (1,83; 2.56) | <0.001 |
| Si | ------------- | |  | Referencia | |  | Referencia | |  | Referencia | |
| Recibió visita domiciliaria del personal del EESS* |  |  |  |  |  |  |  |  |  |  |  |
| No | ------------- | |  | 0.58 (0.27; 1.27) | 0.176 |  | 0.40 (0.34; 0.47) | <0.001 |  | 0.60 (0.08; 4.69) | 0.625 |
| Si | ------------- | |  | Referencia | |  | Referencia | |  | Referencia | |
| Participa en tomar decisiones clave en el hogar* |  |  |  |  |  |  |  |  |  |  |  |
| No | 1.27 (0.64; 2.51) | 0.488 |  | 1.66 (0.88; 3.14) | 0.120 |  | 1.29 (1.16; 1.42) | <0.001 |  | 1.36 (1.08; 1.71) | 0.009 |
| Si | Referencia | |  | Referencia | |  | Referencia | |  | Referencia | |
| Hogar beneficiario del programa social de transferencias condicionadas (JUNTOS)* |  |  |  |  |  |  |  |  |  |  |  |
| No | Referencia | |  | Referencia | |  | Referencia | |  | Referencia | |
| Si | 1.13 (0.28; 4.61) | 0.861 |  | 1.14 (0.4; 3.26) | 0.812 |  | 1.51 (0.97; 2.34) | 0.068 |  | 0.44 (0.21; 0.89) | 0.023 |
| Hogar con seguridad alimentaria (ELCSA)* |  |  |  |  |  |  |  |  |  |  |  |
| No | 0.9 (0.85; 0.96) | <0.001 |  | 0.88 (0.6; 1.28) | 0.491 |  | 1.61 (1.29; 1.99) | <0.001 |  | 1.63 (0.98; 2.7) | 0.058 |
| Si | Referencia | |  | Referencia | |  | Referencia | |  | Referencia | |
| Número de miembros en el hogar | 1.01 (0.98; 1.05) | 0.475 |  | 1.06 (0.96; 1.17) | 0.256 |  | 1.02 (1.01; 1.04) | 0.005 |  | 1 (0.87; 1.15) | 0.985 |
| Puntaje de conocimientos sobre nutrición* | 1 (0.94; 1.05) | 0.884 |  | 1.01 (0.96; 1.05) | 0.835 |  | 0.88 (0.72; 1.07) | 0.205 |  | 1.1 (0.94; 1.3) | 0.233 |
| Fuente de agua del hogar* |  |  |  |  |  |  |  |  |  |  |  |
| No segura | 0.91 (0.67; 1.23) | 0.521 |  | 0.77 (0.77; 0.77) | <0.001 |  | 0.6 (0.1; 3.59) | 0.576 |  | ------------- | |
| Segura | Referencia | |  | Referencia | |  | Referencia | |  | ------------- | |
| Combustible principal para cocinar* |  |  |  |  |  |  |  |  |  |  |  |
| Muy contaminantes | 1.48 (1.1; 1.98) | 0.009 |  | 1.06 (0.51; 2.19) | 0.878 |  | 0.95 (0.34; 2.67) | 0.927 |  | 2.22 (1.34; 3.67) | 0.002 |
| Menos contaminantes | Referencia |  |  | Referencia |  |  | Referencia |  |  | Referencia |  |
| RPcruda: Razón de prevalencia cruda, IC 95%: Intervalo de Confianza al 95%, CRED: Crecimiento y desarrollo. | | | | | | | | | | | |
| ^a^: Modelo omite variable "Niño afiliado a un Seguro de salud" por colinealidad, ^b^: Modelo omite variables "Niño afiliado a un Seguro de salud" "Introducción de alimentos sólidos, semi-sólidos, o suaves a los 6 meses" por colinealidad, ^c^: Modelo omite variables "Niño afiliado a un Seguro de salud" "Introducción de alimentos sólidos, semi-sólidos, o suaves a los 6 meses" "Frecuencia mínima de comidas" "Consumo suplementos de hierro" por colinealidad, ^d^: Modelo omite variables "Niño afiliado a un Seguro de salud" "Introducción de alimentos sólidos, semi-sólidos, o suaves a los 6 meses" "Frecuencia mínima de comidas" "Consumo suplementos de hierro" "Fuente de agua del hogar" por colinealidad | | | | | | | | | | | |

**Tabla S4: Modelos ajustados para la determinación de factores asociados a la presencia de DCI según análisis multivariado en las 4 fases de la cohorte de niños menores de 1 año en Huancavelica y Loreto, Perú.**

|  | **03 meses^a^** | |  | **06 meses^b^** | |  | **09 meses^c^** | |  | **12 meses^d^** | |
| --- | --- | --- | --- | --- | --- | --- | --- | --- | --- | --- | --- |
|  | **RP ajust (IC 95%)** | **Valor P** |  | **RP ajust (IC 95%)** | **Valor P** |  | **RP ajust (IC 95%)** | **Valor P** |  | **RP ajust (IC 95%)** | **Valor P** |
| **CARACTERÍSTICAS DE LOS NIÑOS** |  |  |  |  |  |  |  |  |  |  |  |
| Sexo |  |  |  |  |  |  |  |  |  |  |  |
| Hombre | ------------- | |  | 3.26 (0.87; 12.26) | 0.080 |  | 1.78 (1.7; 1.86) | <0.001 |  | 1.2 (0.99; 1.44) | 0.059 |
| Mujer | ------------- | |  | Referencia | |  | Referencia | |  | Referencia | |
| Niños con Controles CRED completos* |  |  |  |  |  |  |  |  |  |  |  |
| No | 0.98 (0.89; 1.07) | 0.620 |  | 0.39 (0.25; 0.6) | <0.001 |  | ------------- | |  | ------------- | |
| Si | Referencia | |  | Referencia | |  | ------------- | |  | ------------- | |
| Vacunas completas para la edad del niño(a)* |  |  |  |  |  |  |  |  |  |  |  |
| No | 0.78 (0.54; 1.12) | 0.177 |  | ------------- | |  | ------------- | |  | ------------- | |
| Si | Referencia | |  | ------------- | |  | ------------- | |  | ------------- | |
| Niños con episodios de diarrea* |  |  |  |  |  |  |  |  |  |  |  |
| No | Referencia | |  | Referencia | |  | ------------- | |  | Referencia | |
| Si | 1.44 (0.83; 2.51) | 0.195 |  | 1.7 (1; 2.92) | 0.052 |  | ------------- | |  | 0.61 (0.43; 0.87) | 0.007 |
| Episodios de tos* |  |  |  |  |  |  |  |  |  |  |  |
| No | Referencia | |  | Referencia | |  | Referencia | |  | ------------- | |
| Si | 1.48 (1.09; 2.01) | 0.013 |  | 1.31 (0.97; 1.78) | 0.078 |  | 0.88 (0.64; 1.22) | 0.438 |  | ------------- | |
| Amamantados en la primera hora de nacido |  |  |  |  |  |  |  |  |  |  |  |
| No | ------------- | |  | 0.8 (0.73; 0.87) | <0.001 |  | 0.66 (0.63; 0.68) | <0.001 |  | ------------- | |
| Si | ------------- | |  | Referencia | |  | Referencia | |  | ------------- | |
| Lactancia materna exclusiva para la edad o hasta el sexto mes* |  |  |  |  |  |  |  |  |  |  |  |
| No | ------------- | |  | ------------- | |  | 0.49 (0.46; 0.52) | <0.001 |  | 0.69 (0.47; 1.01) | 0.055 |
| Si | ------------- | |  | ------------- | |  | Referencia | |  | Referencia | |
| Uso de biberón |  |  |  |  |  |  |  |  |  |  |  |
| No | ------------- | |  | Referencia | |  | Referencia | |  | ------------- | |
| Si | ------------- | |  | 1.83 (1.11; 3) | 0.017 |  | 0.96 (0.56; 1.63) | 0.874 |  | ------------- | |
| Diversidad mínima de alimentos |  |  |  |  |  |  |  |  |  |  |  |
| No | ------------- | |  | ------------- | |  | ------------- | |  | 0.39 (0.11; 1.44) | 0.158 |
| Si | ------------- | |  | ------------- | |  | ------------- | |  | Referencia | |
| Anemia (<11mg/dL Hemoglobina) |  |  |  |  |  |  |  |  |  |  |  |
| No | ------------- | |  | ------------- | |  | ------------- | |  | Referencia | |
| Si | ------------- | |  | ------------- | |  | ------------- | |  | 0.76 (0.68; 0.86) | <0.001 |
| **CARACTERÍSTICAS DE LAS MADRES Y DEL HOGAR** |  |  |  |  |  |  |  |  |  |  |  |
| Peso de la madre pregestacional | 0.96 (0.95; 0.97) | <0.001 |  | ------------- | |  | ------------- | |  | ------------- | |
| Lengua materna |  |  |  |  |  |  |  |  |  |  |  |
| Otra | 1.1 (0.98; 1.23) | 0.092 |  | 2.02 (1.79; 2.27) | <0.001 |  | 1.14 (0.26; 4.99) | 0.858 |  | 1.84 (1.31; 2.58) | <0.001 |
| Castellano | Referencia | |  | Referencia | |  | Referencia | |  | Referencia | |
| Nivel educativo materno alcanzado |  |  |  |  |  |  |  |  |  |  |  |
| Menos de primaria | ------------- | |  | 0.89 (0.57; 1.38) | 0.592 |  | 2.02 (0.11; 36.37) | 0.634 |  | 1.67 (0.67; 4.2) | 0.273 |
| Secundaria | ------------- | |  | 1.3 (0.6; 2.84) | 0.508 |  | 2.12 (0.27; 16.8) | 0.477 |  | 0.95 (0.86; 1.03) | 0.214 |
| Superior | ------------- | |  | Referencia | |  | Referencia | |  | Referencia | |
| Madre trabajó y/o estudió * |  |  |  |  |  |  |  |  |  |  |  |
| No | Referencia | |  | ------------- | |  | Referencia | |  | Referencia | |
| Si | 1.43 (1.09; 1.87) | 0.010 |  | ------------- | |  | 0.5 (0.18; 1.43) | 0.198 |  | 1.77 (1.09; 2.88) | 0.021 |
| Madre con 3 hijos o más |  |  |  |  |  |  |  |  |  |  |  |
| No | Referencia | |  | ------------- | |  | Referencia | |  | ------------- | |
| Si | 1.95 (1.8; 2.12) | <0.001 |  | ------------- | |  | 1.34 (0.89; 2.02) | 0.157 |  | ------------- | |
| Madres con 6 o más asistencia a controles prenatales |  |  |  |  |  |  |  |  |  |  |  |
| No | ------------- | |  | Referencia | |  | Referencia | |  | ------------- | |
| Si | ------------- | |  | 0.44 (0.42; 0.45) | <0.001 |  | 1.16 (0.85; 1.58) | 0.350 |  | ------------- | |
| Anemia pregestacional |  |  |  |  |  |  |  |  |  |  |  |
| No | ------------- | |  | ------------- | |  | ------------- | |  | Referencia | |
| Si | ------------- | |  | ------------- | |  | ------------- | |  | 0.78 (0.54; 1.14) | 0.201 |
| Recibió sesión alimentaria demostrativa en EESS* |  |  |  |  |  |  |  |  |  |  |  |
| No | ------------- | |  | 0.83 (0.78; 0.88) | <0.001 |  | ------------- | |  | 2 (1.84; 2.17) | <0.001 |
| Si | ------------- | |  | Referencia | |  | ------------- | |  | Referencia | |
| Participa en tomar decisiones clave en el hogar* |  |  |  |  |  |  |  |  |  |  |  |
| No | ------------- | |  | 1.87 (0.89; 3.9) | 0.097 |  | 1.32 (1.29; 1.35) | <0.001 |  | 1.59 (1.51; 1.67) | <0.001 |
| Si | ------------- | |  | Referencia | |  | Referencia | |  | Referencia | |
| Hogar beneficiario del programa social de transferencias condicionadas (JUNTOS)* |  |  |  |  |  |  |  |  |  |  |  |
| No | ------------- | |  | ------------- | |  | Referencia | |  | Referencia | |
| Si | ------------- | |  | ------------- | |  | 1.12 (0.63; 2.02) | 0.699 |  | 0.3 (0.14; 0.67) | 0.003 |
| Hogar con seguridad alimentaria (ELCSA)* |  |  |  |  |  |  |  |  |  |  |  |
| No | 0.77 (0.72; 0.82) | <0.001 |  | ------------- | |  | 1.49 (1.2; 1.85) | <0.001 |  | 1.9 (0.3; 12.21) | 0.499 |
| Si | Referencia | |  | ------------- | |  | Referencia | |  | Referencia | |
| Número de miembros en el hogar |  |  |  |  |  |  | 1 (0.89; 1.12) | 0.980 |  | ------------- | |
| Fuente de agua del hogar* |  |  |  |  |  |  |  |  |  |  |  |
| No segura | ------------- | |  | 0.95 (0.87; 1.04) | 0.300 |  | ------------- | |  | ------------- | |
| Segura | ------------- | |  | Referencia | |  | ------------- | |  | ------------- | |
| Combustible principal para cocinar* |  |  |  |  |  |  |  |  |  |  |  |
| Muy contaminantes | 1.28 (0.87; 1.87) | 0.210 |  | ------------- | |  | ------------- | |  | 2.3 (1.05; 5.02) | 0.037 |
| Menos contaminantes | Referencia | |  | ------------- | |  | ------------- | |  | Referencia | |
| RP ajust: Razón de prevalencia ajustada, IC 95%: Intervalo de Confianza al 95%, CRED: Crecimiento y desarrollo. | | | | | | | | | | | |
| ^a^: Modelo omite variable "Edad de la madre" por multicolinealidad, ^b^: Modelo omite variables "Talla de la madre" "Peso de la madre" "Recibió visita domiciliaria del personal del EESS" por multicolinealidad, ^c^: Modelo omite variables "Edad de la madre" "Talla de la madre" "Peso de la madre" "Recibió sesión alimentaria demostrativa en EESS" "Recibió visita domiciliaria del personal del EESS" por multicolinealidad, ^d^: Modelo omite variables "Vacunas completas para la edad del niño(a)" "Talla de la madre" por multicolinealidad | | | | | | | | | | | |
