# Supplementary material for: Factors associated with childhood chronic malnutrition during the first 12 months of life in children from a peruvian cohort
Source: Rev Peru Med Exp Salud Publica. 2025 Mar 18;42(1):14–27. doi: 10.17843/rpmesp.2025.421.13662 (PMC12176027; doi:10.17843/rpmesp.2025.421.13662)
Supplement: Translation

# Translation: Factores asociados a la desnutrición crónica infantil durante los primeros 12 meses en niños de una cohorte de Perú

**Authors:** Bladimir Morales-Cahuancama, Elena Gonzales-Achuy, Gilmer Solis-Sánchez, Cinthia Quispe-Gala, William Bautista-Olortegui, Gabriela Santos-Antonio, Paúl Hinojosa, Juan Pablo Aparco

**Affiliations:** 1 Centro Nacional de Alimentación, Nutrición y Vida Saludable, Instituto Nacional de Salud, Lima, Perú.; 2 Programa Académico de Nutrición y Dietética, Facultad de ciencias de la salud, Universidad Peruana de Ciencias Aplicadas, Lima, Perú.

**Keywords:** Desnutrición Infantil, Talla Baja, Infantes, Cohorte, Talla Materna, Países de Bajo y Medianos Ingresos

## Abstract

**Objetivo.:**

Determinar los factores asociados a la desnutrición crónica infantil (DCI) en dos regiones peruanas: Huancavelica y Loreto.

**Materiales y métodos:**

. Se realizó un análisis a cuatro evaluaciones transversales repetidas anidadas en una cohorte prospectiva de niños atendidos en establecimientos de salud de primer nivel de atención. Se recopilaron datos sobre características del niño, la madre y el hogar, incluyendo información antropométrica, lactancia, alimentación complementaria, y condiciones socioeconómicas. A través de un modelo lineal generalizado se calculó las razones de prevalencias (RP) por cada una de las cuatro evaluaciones trimestrales de la cohorte (3°, 6°, 9° y 12° meses).

**Resultados.:**

Factores como sexo masculino, uso de biberón y empleo de combustibles contaminantes se asociaron con mayor riesgo de DCI, mientras que recibir seis o más controles prenatales y ser beneficiario del programa JUNTOS actuaron como factores protectores. Las madres con lengua materna distinta al castellano, que trabajaban o estudiaban, o con limitada participación en las decisiones familiares, también presentaron mayor riesgo de DCI. Además, se identificaron asociaciones inesperadas, como la inseguridad alimentaria actuando como factor protector, reflejando posibles sesgos o efectos confusores.

**Conclusiones.:**

Los hallazgos resaltan la importancia de abordar las desigualdades en la atención prenatal, prácticas alimentarias y participación de las madres en la toma de decisiones del hogar para prevenir la DCI en contextos vulnerables.

## INTRODUCCIÓN

La desnutrición crónica infantil (DCI), afecta al 22% de los niños menores de 5 años en el mundo ^1^. A pesar de mejoras significativas, la DCI sigue siendo un problema de salud pública global ^2^. La DCI tiene consecuencias en el crecimiento físico y el neurodesarrollo de los niños ^3^. Entre las repercusiones inmediatas están el aumento significativo de la mortalidad infantil y una mayor susceptibilidad a infecciones ^4^. Además, se observan deficiencias motoras, cognitivas, socioemocionales y conductuales en el neurodesarrollo infantil ^5^. La DCI es más prevalente en países de ingresos bajos y medios (PIBM), afectando el capital humano de la población y sumergiendo al PIBM en un ciclo pernicioso de pobreza ^6^.

En el Perú, la disminución de la DCI muestra una desaceleración en los últimos años ^7^. A nivel subnacional las regiones como Huancavelica y Loreto presentan prevalencias de DCI superiores al promedio nacional ^8^. A pesar de los esfuerzos por identificar los factores asociados a la DCI (socioeconómicos y de acceso a los servicios de salud), la mayoría de estudios son transversales y abordan poblaciones con amplios rangos de edad (<5 años), o son análisis secundarios de encuestas que no recopilan toda la información necesaria de los factores que estarían asociados a la DCI ^9^^,^^10^.

Existen pocos estudios longitudinales desarrollados en PIBM que evalúen los efectos de variables del hogar, de la madre y el cuidado del niño ^11^^-^^13^; sobre todo, en el primer año de edad, donde el crecimiento es acelerado y la alimentación varía desde la ingesta de leche materna al consumo de alimentos sólidos. Además, ocurren las primeras infecciones en el organismo ^14^. Contrario a la percepción generada por estudios transversales de que la DCI se presentaba a mayor edad, un estudio de 32 cohortes longitudinales reveló que el inicio de la DCI ocurre principalmente en los primeros meses de vida ^15^. Esto resalta la importancia de los estudios longitudinales para mostrar que la DCI no solo es acumulativa, sino que comienza a desarrollarse tempranamente; además, permite identificar los determinantes de la DCI que varían en el primer año de vida, brindando una comprensión más dinámica de los factores que contribuyen a su desarrollo.

Dada la diversidad socioeconómica, cultural y biológica en el Perú, y los complejos cambios en el primer año de vida, es necesario investigar la etiología de la DCI a nivel local para implementar intervenciones efectivas. Por tanto, el objetivo del estudio fue identificar los factores asociados a la DCI durante el primer año de vida, a través de evaluaciones transversales trimestrales en una cohorte prospectiva de dos regiones del Perú. Este enfoque metodológico considera la heterogeneidad de las características de alimentación y estilos de vida a lo largo del periodo de estudio.

**MENSAJE CLAVE**

Motivación para realizar el estudio. La desnutrición crónica infantil (DCI) sigue siendo un problema crítico, especialmente en regiones como Huancavelica y Loreto. Aunque existen programas para combatirla, faltaba comprender qué factores específicos afectan a los niños durante su primer año de vida.

Principales hallazgos. Se identificó que ser niño varón, usar biberón y usar combustibles contaminantes aumentan el riesgo de DCI, mientras que asistir a seis o más controles prenatales y recibir apoyo del programa JUNTOS ayudan a prevenirla. Factores como el idioma materno y la participación limitada de las madres también influyen.

Implicancias para la salud pública. Estos hallazgos destacan la necesidad de políticas que fortalezcan la atención prenatal, promuevan prácticas alimentarias saludables y empoderen a las madres en la toma de decisiones.

## MATERIALES Y MÉTODOS

### Diseño del estudio

Estudio observacional con evaluaciones transversales repetidas, anidadas en una cohorte prospectiva fija de niños peruanos, desde los 3 hasta los 12 meses de edad. Se realizaron cuatro evaluaciones trimestrales mediante visitas domiciliarias programadas desde que cumplía el mes de evaluación hasta los 15 días posteriores. En cada fase se excluyeron a los niños que fueron diagnosticados con DCI en la evaluación, permitiendo así centrarse en niños potencialmente sanos. El estudio se realizó en dos regiones: Huancavelica, ubicada en la sierra central a altitudes de 2661 hasta 4274 m s. n. m.; y Loreto, situada en una llanura selvática al noreste del país.

Las evaluaciones transversales del estudio fueron analizadas de manera independiente a manera de estratificación de las observaciones, este enfoque permite identificar las características de los niños a los 3°, 6°, 9° y 12° meses de vida y explorar determinantes asociados a la aparición de la DCI.

### Población y muestra

La población del estudio comprendió niños menores de un año de Huancavelica y Loreto que recibían atención en establecimientos de salud (EESS) del Ministerio de Salud (MINSA). Se calculó una muestra mínima de 660 niños en las dos regiones, y para compensar una posible tasa de atrición de la muestra se enrolaron 1373 niños en total (Material suplementario Figura S1). Detalles adicionales sobre el proceso de muestreo y el impacto de las pérdidas de seguimiento están disponibles en una publicación metodológica previa ^16^.

### Enrolamiento

Inicialmente, se obtuvo el registro de nacimientos en coordinación con los EESS del MINSA correspondientes al primer nivel de atención, ubicadas en zonas urbanas, donde ocurre la mayoría de los nacimientos, lo que facilitó el enrolamiento. Se incluyeron los EESS que, según el registro local de la Estrategia de Salud Reproductiva del MINSA, atendieron un promedio de más de 20 partos mensuales durante los años 2014-2015. A partir de este registro, se elaboró un censo basal que incluyó a todos los recién nacidos desde el 18 de agosto de 2016 en ambas regiones, extendiéndose hasta el 19 de octubre en Loreto y el 23 de noviembre, del mismo año, en Huancavelica.

Una vez construido el registro inicial, el enrolamiento se llevó a cabo en una visita domiciliaria durante el primer mes de vida del niño, entre agosto y diciembre del 2016. Se excluyeron niños de embarazos múltiples, con malformaciones congénitas, prematuros o que nacieron con baja talla ^16^.

### Seguimiento

Cada una de las evaluaciones transversales de la cohorte se llevó a cabo mediante visitas domiciliarias, realizadas por personal del estudio previamente capacitado. Las pérdidas en el seguimiento se debieron principalmente a la migración o mudanza de las familias, así como al rechazo a continuar participando en el estudio ^16^. A los encuestadores se les indicó realizar hasta tres intentos adicionales de contacto en caso de no encontrar a la familia, aplicando el criterio de regresar al domicilio en diferentes horarios para aumentar la probabilidad de completar las evaluaciones.

### Variables

La variable de interés fue la presencia de DCI en cada fase de la cohorte, determinada mediante los puntajes Z de talla para la edad menores de -2 desviaciones estándar por debajo de la mediana del estándar de crecimiento infantil para edad y sexo. En cada momento de evaluación se recogió información de covariables agrupadas según: (a) características de la salud y cuidados del niño, (b) características de la salud y conocimientos de la madre, y (c) características del hogar.

Las características de la salud y cuidados del niño comprendieron aspectos autorreportados por la madre tales como: (a) información de lactancia materna: inicio (en la primera hora de nacido), lactancia materna exclusiva (solo leche materna al 3º y 6º mes), continuidad de lactancia materna (9º y 12º mes), uso de biberón para consumo de leche u otro liquido (por riesgo de enfermedad al 6º, 9º y 12º mes); (b) información de alimentación complementaria: inicio de introducción de alimentos (6º mes), frecuencia mínima de alimentos (9º y 12º mes), diversidad mínima de alimentos (9º y 12º mes); (c) control de crecimiento y desarrollo (CRED), según esquema del MINSA: número de CRED completos para la edad, verificado en carné o referido por la madre (3º, 6º, 9º y 12º mes), vacunas completas para la edad, verificado en carné (3º, 6º, 9º y 12º mes); (d) Suplementación: diagnóstico de anemia (6º y 12º mes), consumo de suplementos, referido por la madre (6º, 9º y 12º mes), consumo adecuado de suplemento de hierro (recepción vs. consumo al 6º, 9º y 12º mes); (e) presencia de infecciones: episodios de diarreas agudas (EDA) (3º, 6º, 9º y 12º mes), infección respiratoria (IRA) (3º, 6º, 9º y 12º mes). Asimismo, se contó con datos obtenidos de fuentes primarias como datos antropométricos y de hemoglobina del niño e información de las historias clínicas como el peso y talla al nacer del niño (Material suplementario Tabla S1).

Característica de la salud y conocimiento de la madre: (a) características generales: edad (referida por la madre), madre con 3 hijos o más (sí/no), lengua materna, nivel educativo (primaria o menos, secundaria y superior), trabaja o estudia; (b) información de la gestación: talla materna (medición antropométrica), peso pregestacional (verificado en carné o referido), número de controles prenatal (CPN) (6 o más controles) y anemia gestacional referida por la madre; (c) Información posparto: participación en sesiones demostrativas sobre alimentación complementaria (al 6º, 9º y 12º mes), visitas domiciliarias recibidas del EESS (al 6º, 9º y 12º mes); (d) otras: participación de la madre en decisiones importantes en el hogar (al 3º, 6º, 9º y 12º mes); conocimiento sobre nutrición a través de preguntas de acuerdo a la edad del niño (Material suplementario Tabla S1).

Características del hogar del niño: (a) número de miembros en el hogar, (b) tener al menos una necesidad básica insatisfecha (NBI) y (c) si el hogar es beneficiario del programa social de transferencia monetaria condicionada (JUNTOS); sobre la vivienda, se catalogaron como adecuado o inadecuado: (d) la fuente de agua y (e) el combustible para cocinar (Material suplementario Tabla S1).

### Métodos estadísticos

Se excluyeron del análisis los niños con valores faltantes en las variables de estudio según correspondía para cada evaluación trimestral. Se caracterizó la distribución de la muestra para cada una de las variables mediante estadística descriptiva, empleando frecuencias y porcentajes para variables categóricas, y media con desviación estándar para las variables numéricas.

La diferencia de la proporción de DCI según las variables estudiadas se evaluó mediante prueba Chi Cuadrado de Pearson y Exacta de Fisher (según regla de Cochran); así también, se comparó las mediciones de las variables numéricas entre quienes tenían o no DCI mediante prueba T de Student para muestras independientes y la U de Mann-Whitney (según normalidad de la distribución).

Para la identificación de factores asociados a la DCI se aplicó un modelo lineal generalizado de familia binomial con enlace Log y opción *cluster* por región (Huancavelica y Loreto) para corregir la variabilidad del origen de los niños; este modelamiento permitió calcular razones de prevalencia (RP) de las variables en cada evaluación trimestral; en donde, aquellas variables que obtuvieron un valor de p menor que 0,2 en el modelo crudo fueron incluidos en el modelo ajustado, siguiendo un criterio de ajuste estadístico ^17^, fueron excluidas las variables que presentaron colinealidad en los modelos crudos y multicolinealidad en el modelo ajustado al encontrar puntuaciones de valor de inflación de la varianza mayor de 10.

Los análisis estadísticos se realizaron de manera estratificada por cada evaluación transversal, para lo cual se empleó el programa estadístico STATA versión 17.0 (Stata Corporation, USA) considerando un nivel de significancia de 0,05.

Además, considerando que cada momento evaluado comprende submuestras de diferentes tamaños por incluir solo niños susceptibles al desenlace, se evaluó la potencia de cada modelo de manera independiente usando el programa G*Power versión 3.1.9.6 (Universisät Kiel, Alemania). En cada una de las cuatro evaluaciones la potencia estadística (1-b) fue de 80%.

### Aspectos éticos

El estudio contó con la aprobación del Comité Institucional de Ética en Investigación del Instituto Nacional de Salud bajo código de protocolo: OI-044-15.

## RESULTADOS

Se enrolaron 1373 niños en Huancavelica y Loreto. Tras el control de calidad de la base de datos se excluyeron algunos participantes, resultando en 968 encuestas incluidas para el análisis a los 3 meses y 513 niños a los 12 meses (Material suplementario Figura S1).

Respecto a las características de los niños; se observó una disminución en la proporción de niños con CRED y vacunas completas a medida que avanzaba la edad, mientras que las EDA e IRA fueron aumentando. En relación con la alimentación infantil, el 39,2% de niños de 6 meses tuvo LME; el uso de biberón se incrementó considerablemente en los meses 9° y 12°, casi todos los niños tuvieron lactancia materna continua. La prevalencia de anemia fue alta (>49%) en la segunda y cuarta evaluación; mientras que la prevalencia de desnutrición crónica fue baja en todas las evaluaciones (Tabla 1).

**Tabla 1:** Características de los niños incluidos en cada evaluación en la cohorte de niños menores de un año. Huancavelica y Loreto, Perú, 2016.

Variables		3 meses	6 meses	9 meses	12 meses
		N= 968	N= 814	N= 685	N =513
		n (%)	n (%)	n (%)	n (%)
Región					
	Huancavelica	407 (42,1)	318 (39,1)	286 (41,8)	219 (42,7)
	Loreto	561 (57,9)	496 (60,9)	399 (58,2)	294 (57,3)
Sexo					
	Hombre	483 (49,9)	396 (48,6)	327 (47,7)	229 (44,6)
	Mujer	485 (50,1)	418 (51,4)	358 (52,3)	284 (55,4)
Niño afiliado a un Seguro de salud ^a^					
	No	7 (0,7)	8 (1,0)	6 (0,9)	11 (2,1)
	Si	961 (99,3)	806 (99,0)	679 (99,1)	502 (97,9)
Niños con Controles CRED completos ^a^					
	No	286 (29,5)	252 (31,0)	249 (36,4)	240 (46,8)
	Si	682 (70,5)	562 (69,0)	436 (63,6)	273 (53,2)
Niños con vacuna completa para la edad ^a^					
	No	169 (17,5)	425 (52,2)	488 (71,2)	506 (98,6)
	Sí	799 (82,5)	389 (47,8)	197 (28,8)	7 (1,4)
Niños con episodios de diarrea ^a^					
	No	770 (79,5)	601 (73,8)	422 (61,6)	314 (61,2)
	Sí	198 (20,5)	213 (26,2)	263 (38,4)	199 (38,8)
Niños con episodios de tos ^a^					
	No	508 (52,5)	387 (47,5)	182 (26,6)	176 (34,3)
	Sí	460 (47,5)	427 (52,5)	503 (73,4)	337 (65,7)
Amamantados en la primera hora de nacido ^a^					
	No	407 (42,0)	343 (42,1)	288 (42,0)	223 (43,5)
	Sí	561 (58,0)	471 (57,9)	397 (58,0	290 (56,5)
Lactancia materna exclusiva para la edad o hasta el sexto mes ^a^					
	No	283 (29,2)	495 (60,8)	405 (59,1)	301 (58,7)
	Sí	685 (70,8)	319 (39,2)	280 (40,9)	212 (41,3)
Uso de biberón ^a^					
	No	866 (89,5)	663 (81,4)	382 (55,8)	298 (58,1)
	Sí	102 (10,5)	151 (18,6)	303 (44,2)	215 (41,9)
Introducción de alimentos sólidos, semisólidos, o suaves a los 6 meses ^a^					
	No	--	23 (2,8)	18 (2,6)	14 (2,7)
	Sí	--	791 (97,2)	667 (97,4)	499 (97,3)
Lactancia materna continua ^a^					
	No	--	--	25 (3,6)	38 (7,4)
	Sí	--	--	660 (96,4)	475 (92,6)
Diversidad mínima de alimentos ^a^					
	No	--	--	136 (19,9)	55 (10,7)
	Sí	--	--	549 (80,1)	458 (89,3)
Frecuencia mínima de comidas ^a^					
	No	--	--	16 (2,3)	3 (0,6)
	Sí	--	--	669 (97,7)	510 (99,4)
Consumo de suplementos de hierro ^a^					
	No	--	107 (13,1)	0 (0,0)	0 (0,0)
	Sí	--	707 (86,9)	685 (100)	513 (100)
Consumo diario de suplementos de hierro ^a^					
	No	--	--	513 (74,9)	324 (63,2)
	Sí	--	--	172 (25,1)	189 (36,8)
Anemia (<11mg/dL de hemoglobina) ^a^					
	No	--	415 (51,0)	--	240 (46,8)
	Sí	--	399 (49,0)	--	273 (53,2)
Desnutrición crónica ^a^					
	No	928 (95,9)	777 (95,5)	646 (94,3)	491 (95,7)
	Sí	40 (4,1)	37 (4,5)	39 (5,7)	22 (4,3)

N: total de sujetos en la muestra, n: cantidad de sujetos en la categoría, %: porcentaje de sujetos en la categoría

a Durante los tres meses previos a la encuesta.

En relación a las características de las madres y del hogar (Tabla 2), la mayoría tuvo como lengua materna al castellano y alcanzó el nivel educativo secundario. La proporción de madres que compartía el tiempo de cuidado del niño con su trabajo o estudio fue aumentando conforme el seguimiento transcurría. La cobertura de las sesiones demostrativas y las visitas domiciliarias, en los últimos tres meses previos a cada evaluación, fue baja (<9,4%). Asimismo, la mayoría de madres participaron en las decisiones clave en el hogar tales como llevar al niño al EESS y actividades relacionadas con su alimentación. Respecto a las características de los hogares una proporción considerable no tuvo seguridad alimentaria; la mayor proporción de viviendas contaban con fuente de agua segura y combustible adecuado para cocinar. Los resultados completos de las características de niños, madres y hogar se presentan en la tabla S2 del material suplementario.

**Tabla 2:** Características de las madres y del hogar incluidos en cada evaluación de la cohorte de niños menores de un año en Huancavelica y Loreto, Perú 2016.

Variables		3 meses	6 meses	9 meses	12 meses
		N= 968	N= 814	N= 685	N =513
		n (%)	n (%)	n (%)	n (%)
	Edad de la madre (media ± DE)	26,1±6,6	26,1±6,7	26,4±6,7	27,5±6,8
	Peso pregestacional de la madre (media ± DE)	57,1±9,4	57,3±9,6	57,9±9,6	58,2±9,8
	Talla de la madre (media ± DE)	151,3±5,0^b^	151,3±5,0	151,6±5,0	151,7±5,0
Lengua materna					
	Otra	235 (24,3)	179 (22,0)	168 (24,5)	138 (26,9)
	Castellano	733 (75,7)	635 (78,0)	517 (75,5)	375 (73,1)
Nivel educativo materno alcanzado					
	Primaria o menos	235 (24,3)	200 (24,6)	167 (24,4)	135 (26,3)
	Secundaria	544 (56,2)	453 (55,7)	379 (55,3)	275 (53,6)
	Superior	189 (19,5)	161 (19,8)	139 (20,3)	103 (20,1)
Madre trabajó y/o estudió ^a^					
	No	622 (64,3)	446 (54,8)	291 (42,5)	211 (41,1)
	Sí	346 (35,7)	368 (45,2)	394 (57,5)	302 (58,9)
Madre con 3 hijos o más					
	No	786 (81,2)	662 (81,3)	549 (80,1)	414 (80,7)
	Sí	182 (18,8)	152 (18,7)	136 (19,9)	99 (19,3)
Madre con 6 o más asistencias a controles prenatales					
	No	167 (17,3)	130 (16,0)	102 (14,9)	67 (13,1)
	Sí	801 (82,7)	684 (84,0)	583 (85,1)	446 (86,9)
Anemia pregestacional					
	No	586 (60,5)	496 (60,9)	416 (60,7)	314 (61,2)
	Sí	382 (39,5)	318 (39,1)	269 (39,3)	199 (38,8)
Participación en sesiones demostrativas en EESS ^a^					
	No	--	783 (96,2)	635 (92,7)	465 (90,6)
	Sí	--	31 (3,8)	50 (7,3)	48 (9,4)
Recibió visita domiciliaria del personal del EESS ^a^					
	No	--	801 (98,4)	655 (95,6)	484 (94,3)
	Sí	--	13 (1,6)	30 (4,4)	29 (5,7)
Participa en tomar decisiones clave en el hogar ^a^					
	No	450 (46,5)	296 (36,4)	224 (32,7)	195 (38,0)
	Sí	518 (53,5)	518 (63,6)	461 (67,3)	318 (62,0)
Hogar beneficiario del programa social de transferencias condicionadas (JUNTOS) ^a^					
	No	748 (77,3)	655 (80,5)	529 (77,2)	377 (73,5)
	Sí	220 (22,7)	159 (19,5)	156 (22,8)	136 (26,5)
Hogar con seguridad alimentaria (ELCSA) ^a^					
	No	721 (74,5)	531 (65,2)	380 (55,5)	241 (47,0)
	Sí	247 (25,5)	283 (34,8)	305 (44,5)	272 (53,0)
Hogar con al menos una NBI ^a^					
	No	0 (0,0)	0 (0,0)	0 (0,0)	0 (0,0)
	Sí	968 (100)	814 (100)	685 (100)	513 (100)
Número de miembros en el hogar		6,0 ±2,4	5,9 ±2,4	6,1 ±2,3	5,8 ±2,3
Puntaje de conocimientos sobre nutrición ^a^		3,3 ±1,0	4,4 ±1,8	3,7 ±1,9	2,4 ±1,1
Fuente de agua del hogar ^a^					
	No segura	311 (32,1)	265 (32,6)	206 (30,1)	150 (29,2)
	Segura	657 (67,9)	549 (67,4)	479 (69,9)	363 (70,8)
Combustible principal para cocinar ^a^					
	Muy contaminantes	238 (24,6)	211 (25,9)	182 (26,6)	122 (23,8)
	Menos contaminantes	730 (75,4)	603 (74,1)	503 (73,4)	391 (76,2)

CRED: controles de crecimiento y desarrollo, EESS: establecimiento de salud, NBI: necesidad básica insatisfecha, N: total de sujetos en la muestra, n: cantidad de sujetos en la categoría, %: porcentaje de sujetos en la categoría, DE: desviación estándar de la variable en la muestra, ELCSA: Escala Latinoamericana y Caribeña de Seguridad Alimentaria.

a Durante los tres meses previos a la encuesta, ^b^ medido en 846 mujeres.

En la tabla 3 se presenta el análisis bivariado para cada evaluación transversal. Cabe señalar que sólo se muestran aquellas variables en las que se obtuvo diferencias significativas en al menos una de las evaluaciones. A los seis meses se encontró diferencias significativas en las variables de CRED completos (valor de p=0,047), alimentación usando biberón (valor de p=0,026) y sexo del niño (valor de p=0,001). A los 9 meses hubo diferencias significativas con las madres que trabajan o estudian (valor de p=0,032) y recibieron alguna sesión demostrativa en los meses previos (valor de p=0,005). A los 12 meses sólo hubo diferencias en la variable fuente de agua del hogar (valor de p=0,002). Los resultados completos se presentan en la Tabla S3 del material suplementario.

**Tabla 3:** Factores asociados (categóricos) a la DCI según análisis bivariado en las cuatro evaluaciones de la cohorte de niños menores de un año en Huancavelica y Loreto, Perú 2016.

Variables		DCI a los 3 meses		DCI a los 6 meses		DCI a los 9 meses		DCI a los 12 meses	
		n (%)	Valor de p	n (%)	Valor de p	n (%)	Valor de p	n (%)	Valor de p
Características de los niños									
Región									
	Huancavelica	19 (4,7)	0,475 ^a^	16 (5,0)	0,594 ^a^	24 (8,4)	0,010 ^a^	13 (5,9)	0,112 ^a^
	Loreto	21 (3,7)		21 (4,2)		15 (3,8)		9 (3,1)	
Sexo									
	Hombre	24 (5,0)	0,192 ^a^	28 (7,1)	0,001 ^a^	24 (7,3)	0,076 ^a^	11 (4,8)	0,605 ^a^
	Mujer	16 (3,3)		9 (2,2)		15 (4,2)		11 (3,9)	
Niño afiliado a un Seguro de salud									
	No	0 (0,0)	1,000 ^b^	0 (0,0)	1,000 ^b^	0 (0)	1,000 ^b^	1 (9,1)	0,386 ^b^
	Sí	40 (4,2)		37 (4,6)		39 (5,7)		21 (4,2)	
Niños con CRED completos ^c^									
	No	11 (3,9)	0,772 ^a^	6 (2,4)	0,047 ^a^	12 (4,8)	0,456 ^a^	7 (2,9)	0,150 ^a^
	Sí	29 (4,3)		31 (5,5)		27 (6,2)		15 (5,5)	
Vacunas completas para la edad del niño(a) ^c^									
	No	6 (3,6)	0,676 ^a^	20 (4,7)	0,818 ^a^	29 (5,9)	0,658 ^a^	21 (4,2)	0,266 ^b^
	Sí	34 (4,3)		17 (4,4)		10 (5,1)		1 (14,3)	
Niños con episodios de diarrea ^c^									
	No	29 (3,8)	0,259 ^a^	23 (3,8)	0,098 ^a^	24 (5,7)	0,993 ^a^	16 (5,1)	0,257 ^a^
	Sí	11 (5,6)		14 (6,6)		15 (5,7)		6 (3,0)	
Niños con episodio de tos ^c^									
	No	17 (3,4)	0,197 ^a^	16 (4,1)	0,592 ^a^	13 (7,1)	0,325 ^a^	5 (2,8)	0,242 ^a^
	Sí	23 (5,0)		21 (4,9)		26 (5,2)		17 (5,0)	
Amamantados en la primera hora de nacido									
	No	18 (4,4)	0,699 ^a^	13 (3,8)	0,377 ^a^	12 (4,2)	0,142 ^a^	7 (3,1)	0,260 ^a^
	Sí	22 (3,9)		24 (5,1)		27 (6,8)		15 (5,2)	
Lactancia materna exclusiva para la edad o hasta el sexto mes ^c^									
	No	10 (3,5)	0,548 ^a^	25 (5,1)	0,389 ^a^	16 (4)	0,018 ^a^	10 (3,3)	0,198 ^a^
	Sí	30 (4,4)		12 (3,8)		23 (8,2)		12 (5,7)	
Uso de biberón									
	No	35 (4,0)	0,602 ^b^	25 (3,8)	0,026 ^a^	24 (6,3)	0,455 ^a^	14 (4,7)	0,590 ^a^
	Sí	5 (4,9)		12 (8,0)		15 (5)		8 (3,7)	
Introducción de alimentos sólidos, semisólidos, o suaves a los 6 meses									
	No	--	--	0 (0,0)	0,619 ^b^	0 (0)	0,617 ^b^	0 (0,0)	1,000 ^b^
	Sí	--		37 (4,7)		39 (5,9)		22 (4,4)	
Lactancia materna continua									
	No	--	--	--	--	1 (4)	1,000 ^b^	1 (2,6)	1,000 ^b^
	Sí	--		--		38 (5,8)		21 (4,4)	
Diversidad mínima de alimentos									
	No	--	--	--	--	6 (4,4)	0,471 ^a^	1 (1,8)	0,495 ^b^
	Sí	--		--		33 (6)		21 (4,6)	
Frecuencia mínima de comidas									
	No	--	--	--	--	0 (0,0)	1,000 ^b^	0 (0,0)	1,000 ^b^
	Sí	--		--		39 (5,8)		22 (4,3)	
Consumo suplementos de hierro ^c^									
	No	--	--	4 (3,7)	0,807 ^b^	--	NC	--	NC
	Sí	--		33 (4,7)		39 (5,7)		22 (4,3)	
Anemia (<11mg/dL de hemoglobina)									
	No	--	--	19 (4,6)	0,963 ^a^	--	--	15 (4,8)	0,493 ^a^
	Sí	--		18 (4,5)		--		7 (3,5)	
Características de las madres y del hogar									
Lengua materna									
	Otra	11 (4,7)	0,627 ^a^	12 (6,7)	0,116 ^a^	14 (8,3)	0,089 ^a^	9 (6,5)	0,130 ^a^
	Castellano	29 (4,0)		25 (3,9)		25 (4,8)		13 (3,5)	
Nivel educativo materno alcanzado									
	Primaria o menos	13 (5,5)	0,421 ^a^	9 (4,5)	0,587 ^a^	13 (7,8)	0,096 ^a^	8 (5,9)	0,547 ^a^
	Secundaria	19 (3,5)		23 (5,1)		23 (6,1)		10 (3,6)	
	Superior	8 (4,2)		5 (3,1)		3 (2,2)		4 (3,9)	
Madre trabajó y/o estudió ^c^									
	No	22 (3,5)	0,212 ^a^	22 (4,9)	0,559 ^a^	23 (7,9)	0,032 ^a^	6 (2,8)	0,177 ^a^
	Si	18 (5,2)		15 (4,1)		16 (4,1)		16 (5,3)	
Madre con 3 hijos o más									
	No	29 (3,7)	0,150 ^a^	30 (4,5)	0,969 ^a^	28 (5,1)	0,178 ^a^	17 (4,1)	0,592 ^b^
	Sí	11 (6,0)		7 (4,6)		11 (8,1)		5 (5,1)	
Madres con 6 o más asistencia a controles prenatales									
	No	10 (6,0)	0,185 ^a^	9 (6,9)	0,156 ^a^	5 (4,9)	0,708 ^a^	2 (3,0)	0,754 ^b^
	Sí	30 (3,8)		28 (4,1)		34 (5,8)		20 (4,5)	
Anemia pregestacional									
	No	24 (4,1)	0,943 ^a^	25 (5,0)	0,397 ^a^	27 (6,5)	0,263 ^a^	15 (4,8)	0,493 ^a^
	Sí	16 (4,2)		12 (3,8)		12 (4,5)		7 (3,5)	
Participación en sesiones demostrativas en EESS ^c^									
	No	--	--	35 (4,5)	0,647 ^b^	31 (4,9)	0,005 ^b^	21 (4,5)	0,710 ^b^
	Sí	--		2 (6,5)		8 (16)		1 (2,1)	
Recibió visita domiciliaria del personal del EESS ^c^									
	No	--	--	36 (4,5)	0,456 ^b^	35 (5,3)	0,084 ^b^	20 (4,1)	0,357 ^b^
	Sí	--		1 (7,7)		4 (13,3)		2 (6,9)	
Participa en tomar decisiones clave en el hogar ^c^									
	No	21 (4,7)	0,436 ^a^	18 (6,1)	0,112 ^a^	15 (6,7)	0,430 ^a^	10 (5,1)	0,462 ^a^
	Sí	19 (3,7)		19 (3,7)		24 (5,2)		12 (3,8)	
Hogar beneficiario del programa social de transferencias condicionadas (JUNTOS) ^c^									
	No	30 (4,0)	0,726 ^a^	29 (4,4)	0,743 ^a^	27 (5,1)	0,220 ^a^	19 (5,0)	0,162 ^a^
	Sí	10 (4,6)		8 (5,0)		12 (7,7)		3 (2,2)	
Hogar con seguridad alimentaria (ELCSA) ^c^									
	No	29 (4,0)	0,769 ^a^	23 (4,3)	0,688 ^a^	26 (6,8)	0,148 ^a^	13 (5,4)	0,245 ^a^
	Sí	11 (4,5)		14 (5,0)		13 (4,3)		9 (3,3)	
Fuente de agua del hogar ^c^									
	No segura	12 (3,9)	0,768 ^a^	10 (3,8)	0,463 ^a^	8 (3,9)	0,180 ^a^	0 (0,0)	0,002 ^a^
	Segura	28 (4,3)		27 (4,9)		31 (6,5)		22 (6,1)	
Combustible principal para cocinar ^c^									
	Muy contaminantes	13 (5,5)	0,235 ^a^	10 (4,7)	0,875 ^a^	10 (5,5)	0,892 ^a^	9 (7,4)	0,054 ^a^
	Menos contaminantes	27 (3,7)		27 (4,5)		29 (5,8)		13 (3,3)	
Total		40 (4,1)		37 (4,6)		39 (5,7)		22 (4,3)	

a prueba Chi cuadrado de Pearson, ^b^ prueba Exacta de Fisher, ^c^ durante los tres meses previos a la encuesta, DCI: desnutrición crónica infantil. NC: no calculable por ser constante.

Además, en la tabla 4, se muestran todas las variables de tipo cuantitativa que se analizaron en cada evaluación de la cohorte. La variable que tuvo diferencias significativas en más de una oportunidad fue la talla materna (valor de p<0,001). Mientras que el peso materno pregestacional tuvo diferencias en la segunda y tercera evaluación.

**Tabla 4:** Factores asociados (cuantitativos) a la DCI según análisis bivariado en las cuatro evaluaciones de la cohorte de niños menores de un año en Huancavelica y Loreto, Perú 2016.

Variables	3 meses			6 meses			9 meses			12 meses		
	Sin DCI M±DE	Con DCI M±DE	Valor de p	Sin DCI M±DE	Con DCI M±DE	Valor de p	Sin DCI M±DE	Con DCI M±DE	Valor de p	Sin DCI M±DE	Con DCI M±DE	Valor de p
Edad de la madre	26,0 ±6,6	26,4 ±7	0,663 ^b^	26,2 ±6,6	24,7 ±7	0,113 ^b^	26,4 ±6,7	27,4 ±7,6	0,429 ^b^	27,5 ±6,8	27,5 ±6,0	0,873 ^b^
Peso pregestacional de la madre	56,8 ±9,4	54,1 ±8,2	0,058 ^b^	57,5 ±9,6	53,0 ±7,3	0,009 ^b^	58,1 ±9,5	53,5 ±9,4	0,005 ^b^	58,3 ±9,8	54,5 ±8,7	0,153 ^b^
Talla de la madre	151,3 ±5,1	--	NC ^a^	151,5 ±4,9	148,2 ±4,3	<0,001 ^b^	151,8 ±5,0	148,5 ±4,5	<0,001^b^	151,8 ±5	149,6 ±5,3	0,027 ^b^
Número de miembros en el hogar	5,9 ±2,4	6 ±1,9	0,438 ^b^	5,9 ±2,3	6,3 ±2,5	0,357 ^b^	6,1 ±2,3	6,2 ±1,9	0,436 ^b^	5,8 ±2,2	5,8 ±2,6	0,713 ^b^
Puntaje de conocimientos sobre nutrición	3,3 ±0,9	3,3 ±1	0,980 ^c^	4,4 ±1,8	4,4 ±1,5	0,762 ^b^	3,7 ±1,9	3,2 ±1,7	0,114 ^b^	2,4 ±1,1	2,5 ±1,0	0,613 ^c^

DCI: desnutrición crónica infantil, M: media, DE: desviación estándar.

a No calculable por haberse registrado a partir de los 6 meses, ^b^ prueba U de Mann-Whitney^, c^ prueba T de Student para muestras independientes con varianzas iguales.

En la tabla 5 se presentan las RP ajustadas de las variables para cada evaluación trimestral. A los 3 meses, los factores de riesgo asociados a DCI fueron episodios de tos (RP: 1,48. IC95%: 1,09; 2,01. p=0,013), madres con 3 hijos o más (RP: 1,95. IC95%: 1,8; 2,12. p<0,001) y las madres que trabajan o estudian (RP: 1,43. IC95%: 1,09; 1,87. p=0,010). Como factores protectores se identificaron el peso pregestacional de la madre (RP: 0,96. IC95%: 0,95; 0,97. p<0.001) y hogares con inseguridad alimentaria (RP: 0,77. IC95%: 0,72; 0,82. p<0,001). A los 6 meses, se identificaron como factores de riesgo la alimentación con biberón (RP: 1,83. IC95%: 1,11;3. p=0,017) y las madres con lengua materna distinta al castellano (RP: 2,02. IC95%: 1,79; 2,27. p<0,001). Por otro lado, los factores protectores incluyeron no tener CRED completos (RP: 0,39. IC95%: 0,25; 0,6. p<0,001); no amamantar en la primera hora de nacido (RP: 0,8. IC95%: 0,73; 0,87. p<0,001); madres que asistieron a la mayoría de CPN (RP: 0,44. IC95%: 0,42; 0,45. p<0,001) y no haber recibido una sesión demostrativa (RP: 0,83. IC95%: 0,78; 0,88. p<0,001). A los 9 meses, los factores de riesgo identificados fueron el sexo masculino (RP: 1,78. IC95%: 1,70; 1,86. p<0,001), madres que no participan en las decisiones clave del hogar (RP: 1,32. IC95%: 1,29; 1,35. p<0,001) y hogares con inseguridad alimentaria (RP: 1,49. IC95%: 1,20; 1,85. p<0,001). Como factores protectores se encontraron no recibir leche materna en la primera hora de vida (RP: 0,66. IC95%: 0,63; 0,68. p<0,001) y no recibir LME hasta el sexto mes (RP: 0,49. IC95%: 0,46; 0,52. p<0,001). Finalmente, a los 12 meses, los factores de riesgo incluyeron madres con lengua materna distinta al castellano (RP: 1,84. IC95%: 1,31; 2,58. p<0.001), madres que trabajan y/o estudian (RP: 1,77. IC95%: 1,09; 2,88. p=0,021), no haber recibido una sesión demostrativa (RP: 2,00. IC95%: 1,84; 2,17. p<0,001), madres que no participan en las decisiones clave del hogar (RP: 1,59. IC95%: 1,51; 1,67. p<0,001) y uso de combustible muy contaminante (RP: 2,30. IC95%: 1,05; 5,02. p=0,037). Como factores protectores se encontró episodios de diarreas (RP: 0,61. IC95%: 0,43; 0,87. p=0,007), anemia (RP: 0,76. IC95%: 0,68; 0,86. p<0,001) y pertenecer al programa social de transferencias condicionadas JUNTOS (RP: 0,3. IC95%: 0,14; 0,67. p=0,003). Los modelos crudos y ajustados completos se encuentran en las tablas S3 y S4 del material suplementario.

**Tabla 5:** Factores asociados a la desnutrición crónica infantil (DCI) según análisis multivariado en las cuatro evaluaciones de la cohorte de niños menores de un año en Huancavelica y Loreto, Perú.

Características de los niños		3 meses ^a^		6 meses ^b^		9 meses ^c^		12 meses ^d^	
		RPa (IC95%)	Valor de p	RPa (IC95%)	Valor de p	RPa (IC95%)	Valor de p	RPa (IC95%)	Valor de p
Sexo									
	Hombre	--		--		1,78 (1,7; 1,86)	<0,001	--	
	Mujer	--		--		Referencia		--	
Niños con CRED completos									
	No	--		0,39 (0,25; 0,6)	<0,001	--		--	
	Sí	--		Referencia		--		--	
Niños con episodios de diarrea									
	No	--		--		--		Referencia	
	Sí	--		--		--		0,61 (0,43; 0,87)	0,007
Niños con episodio de tos									
	No	Referencia		--		--		--	
	Sí	1,48 (1,09; 2,01)	0,013	--		--		--	
Amamantados en la primera hora de nacido									
	No	--		0,8 (0,73; 0,87)	<0,001	0,66 (0,63; 0,68)	<0,001	--	
	Sí	--		Referencia		Referencia		--	
Lactancia materna exclusiva para la edad o hasta el sexto mes									
	No	--		--		0,49 (0,46; 0,52)	<0,001	--	
	Sí	--		--		Referencia		--	
Uso de biberón									
	No	--		Referencia		--		--	
	Sí	--		1,83 (1,11; 3,0)	0,017	--		--	
Anemia (<11mg/dL Hemoglobina)									
	No	--		--		--		Referencia	
	Sí	--		--		--		0,76 (0,68; 0,86)	<0,001
Características de las madres y del hogar									
Peso pregestacional de la madre		0,96 (0,95; 0,97)	<0,001	--		--		--	
Lengua materna									
	Otra	--		2,02 (1,79; 2,27)	<0,001	--		1,84 (1,31; 2,58)	<0,001
	Castellano	--		Referencia		--		Referencia	
Madre trabajó y/o estudió									
	No	Referencia		--		--		Referencia	
	Sí	1,43 (1,09; 1,87)	0,010	--		--		1,77 (1,09; 2,88)	0,021
Madre con 3 hijos o más									
	No	Referencia		--		--		--	
	Sí	1,95 (1,8; 2,12)	<0,001	--		--		--	
Madres con 6 o más asistencia a controles prenatales									
	No	--		Referencia		--		--	
	Sí	--		0,44 (0,42; 0,45)	<0,001	--		--	
Participó en sesiones demostrativas en EESS									
	No	--		0,83 (0,78; 0,88)	<0,001	--		2 (1,84; 2,17)	<0,001
	Sí	--		Referencia		--		Referencia	
Participa en tomar decisiones clave en el hogar									
	No	--		--		1,32 (1,29; 1,35)	<0,001	1,59 (1,51; 1,67)	<0,001
	Sí	--		--		Referencia		Referencia	
Hogar beneficiario del programa social de transferencias condicionadas (JUNTOS)									
	No	--		--		--		Referencia	
	Sí	--		--		--		0,3 (0,14; 0,67)	0,003
Hogar con seguridad alimentaria (ELCSA)									
	No	0,77 (0,72; 0,82)	<0,001	--		1,49 (1,2; 1,85)	<0,001	--	
	Sí	Referencia		--		Referencia		--	
Combustible principal para cocinar									
	Muy contaminantes	--		--		--		2,3 (1,05; 5,02)	0,037
	Menos contaminantes	--		--		--		Referencia	

RPa: razón de prevalencia ajustada, IC95%: intervalo de confianza al 95%, CRED: controles de crecimiento y desarrollo, EESS: establecimiento de salud.

a Modelo a los tres meses, omite variables: “edad de la madre” por multicolinealidad y ajustado por “niños con controles CRED completos”, “vacunas completas para la edad del niño(a)”, “niños con episodios de diarrea”, “lengua materna” y “combustible principal para cocinar”. Potencia estadística (1-b) de 0,801.

b Modelo a los seis meses, omite variables: “talla de la madre”, “peso de la madre” y “recibió visita domiciliaria del personal del EESS” por multicolinealidad y ajustado por “sexo”, “niños con episodios de diarrea”, “niños con episodio de tos”, “nivel educativo materno alcanzado”, “participa en tomar decisiones clave en el hogar” y “fuente de agua del hogar”. Potencia estadística (1-b) de 0,800.

c Modelo a los nueve meses, omite variables: “edad de la madre”, “talla de la madre”, “peso de la madre”, “participó en sesiones demostrativas en EESS” y “recibió visita domiciliaria del personal del EESS” por multicolinealidad y ajustado por “niños con episodio de tos”, “uso de biberón”, “lengua materna”, “nivel educativo materno alcanzado”, “madre trabajó y/o estudió”, “madre con 3 hijos o más”, “madres con 6 o más asistencia a controles prenatales”, “hogar beneficiario del programa social de transferencias condicionadas (JUNTOS)” y “número de miembros en el hogar”. Potencia estadística (1-b) de 0,803.

d Modelo a los doce meses, omite variables: “vacunas completas para la edad del niño(a)” y “talla de la madre” por multicolinealidad y ajustado por “sexo”, “lactancia materna exclusiva para la edad o hasta el sexto mes”, “diversidad mínima de alimentos”, “nivel educativo materno alcanzado”, “anemia pregestacional” y “hogar con seguridad alimentaria (ELCSA)”. Potencia estadística (1-b) de 0,801.

## DISCUSIÓN

La investigación reveló una variedad de factores asociados a la DCI durante el primer año de vida del niño. Algunos de estos factores manifestaron su influencia en un solo corte transversal como el sexo masculino, uso de biberon y empleo de combustible muy contaminante asociados al riesgo; en tanto que como protectores se encontró que las madres reciban 6 o más CPN y ser beneficiario del programa JUNTOS. Otros factores mostraron su influencia en distintas evaluaciones como madres con lengua materna distinta al castellano, madres que trabajaron o estudiaron durante el primer año de vida del niño y madres que no participan de las decisiones claves del hogar que actuaron como determinante de riesgo. Además, se identificaron factores con efectos controvertidos en relación a servicios destinados a prevenir la DCI, como los CRED, las sesiones demostrativas o la presencia de enfermedades como anemia o diarrea en el niño. Un hallazgo poco explorado en el contexto peruano fue la relación entre la participación de la madre en decisiones clave del hogar y la DCI.

### Factores asociados a la DCI a los 3 meses

A los 3 meses, los episodios de tos se identificaron como un riesgo significativo, en línea con estudios que evidencian cómo las IRA comprometen el estado nutricional al aumentar los requerimientos metabólicos, reducir la ingesta alimentaria y alterar el eje hormonal del crecimiento ^18^^,^^19^. La presencia de tres o más hijos también representó un riesgo, posiblemente debido a la competencia por recursos limitados y la reducción en la atención materna ^20^^,^^21^. De manera similar, las madres que estudiaban o trabajaban mostraron una asociación negativa, lo cual, en contextos vulnerables, podría estar relacionado con menor tiempo para prácticas clave como la lactancia exclusiva y la atención directa al niño ^22^^,^^23^.

Por otro lado, el peso pregestacional materno actuó como factor protector, lo que respalda la importancia de un buen estado nutricional materno para el desarrollo infantil temprano ^24^. Finalmente, la asociación paradójica de la inseguridad alimentaria como factor protector podría reflejar sesgos en su medición o efectos confusores, como la priorización de recursos hacia los miembros más vulnerables del hogar ^25^.

### Factores asociados a la DCI a los 6 meses

La alimentación con biberón se identificó como un factor de riesgo, coincidiendo con estudios que resaltan su papel como foco de infecciones, especialmente en entornos con acceso limitado a agua potable y condiciones de higiene inadecuadas ^26^^,^^27^. Asimismo, el riesgo asociado a madres con lengua materna distinta al castellano refleja disparidades socioeconómicas y barreras en el acceso a servicios de salud y educación, lo cual puede afectar negativamente la nutrición infantil ^28^.

Entre los factores protectores, se observó que la asistencia regular a CPN disminuyó el riesgo de DCI, lo que refuerza la importancia del monitoreo materno durante el embarazo y la capacitación en prácticas de cuidado infantil ^29^. No obstante, resultados aparentemente contradictorios surgieron respecto a la asistencia completa a CRED y a sesiones demostrativas, que se asociaron con un mayor riesgo de DCI. Una posible explicación es que los niños con mayor riesgo de retraso de crecimiento hayan sido identificados tempranamente, generando una mayor asistencia al CRED. Sin embargo, factores subyacentes como vulnerabilidad socioeconómica, dificultades para cumplir recomendaciones y limitaciones de acceso a recursos podrían limitar la efectividad de estas intervenciones.

### Factores asociados a la DCI a los 9 meses

El sexo masculino se asoció a un mayor riesgo de DCI, posiblemente vinculado a diferencias en la atención brindada según el género, lo que coincide con estudios que señalan una mayor vulnerabilidad neonatal y perinatal en varones ^30^.

La baja participación de las madres en decisiones del hogar también fue un factor de riesgo, respaldando investigaciones que asocian la limitada autonomía materna con peores resultados en salud infantil ^31^^,^^32^. Las madres con mayor autonomía tienden a participar activamente en decisiones sobre alimentación y cuidado, lo que favorece prácticas protectoras.

La inseguridad alimentaria en el hogar, otro factor de riesgo refleja las múltiples vías por las cuales la crisis alimentaria afecta el crecimiento infantil. Este fenómeno no solo compromete la disponibilidad de alimentos, sino que también genera estrés familiar, malnutrición materna y alteraciones en prácticas de crianza, lo que agrava el riesgo de DCI ^33^.

Un hallazgo inesperado fue que niños sin LME hasta el sexto mes tenían menos probabilidad de DCI; al respecto un estudio previo en Perú reportó que esta asociación reflejaría causalidad inversa, en tanto que otro estudio internacional también han reportado hallazgos similares ^34^.

### Factores asociados a la DCI a los 12 meses

Al igual que a los 6 meses, los hijos de madres con lengua materna diferente al castellano presentan mayor riesgo de DCI; este hallazgo, poco reportado en artículos a pesar de su disponibilidad en encuestas nacionales, refleja su estrecha asociación con la pobreza multidimensional, ya que está vinculada a la pertenencia a grupos indígenas y a condiciones de exclusión social ^28^. En Perú, estudios han reportado que la prevalencia de DCI en niños indígenas es el doble comparado con niños no indígenas ^35^, lo que subraya la relevancia de esta variable en contextos de desigualdad estructural.

Asimismo, los hijos de madres que trabajaron o estudiaron tenían más riesgo de DCI, posiblemente debido a la competencia con el tiempo dedicado al cuidado infantil. Este resultado coincide con estudios que asocian la doble carga laboral con desenlaces negativos en la salud infantil ^22^.

La no participación de las madres en decisiones claves del hogar reafirma su impacto negativo en el crecimiento infantil, limitando el acceso a recursos y decisiones informadas para el cuidado del niño ^31^^,^^32^.

El uso de combustibles altamente contaminantes, como leña o carbón, también se asoció a mayor riesgo de DCI. Estos combustibles incrementan las infecciones respiratorias y afectan indirectamente el estado nutricional, además de servir como un indicador del nivel socioeconómico del hogar ^36^.

Entre los factores protectores, la asociación negativa de la diarrea y la anemia con la DCI puede deberse a la naturaleza transversal del análisis y un posible sesgo de causalidad reversa, donde los niños afectados recibieron cuidados intensivos que mitigaron el impacto en su crecimiento o su ocurrencia fue muy reciente sin afectar el estado nutricional. Este hallazgo resalta la necesidad de investigar la frecuencia y severidad de estas condiciones en estudios longitudinales para comprender su relación con la DCI.

Por otro lado, pertenecer al programa social «JUNTOS» se identificó como un factor protector clave, posiblemente por facilitar el acceso a servicios como CRED y vacunación completa.

Los resultados de este estudio destacan el primer año de vida como un periodo crítico donde múltiples factores interactúan para influir en la DCI. Esto subraya la importancia de evaluar de manera integral los determinantes sociales, económicos y ambientales en regiones como Huancavelica y Loreto. Vincular el crecimiento infantil con el desarrollo cognitivo y establecer sistemas de seguimiento nominal permitirá identificar riesgos y prevenir su impacto. Estos hallazgos respaldan el diseño de políticas integrales que incluyan servicios de salud, programas de alimentación, apoyo al cuidado infantil, seguridad alimentaria, empoderamiento femenino y mejoras en las condiciones sanitarias del hogar.

El estudio se llevó a cabo con una perspectiva longitudinal, aunque el análisis de los datos fue como cortes transversales, anidado en el sistema de salud público peruano, sin alterar las actividades regulares de los EESS. Esto contrasta con los entornos controlados de otros estudios de seguimiento; lo que permitió la obtención de datos más realistas de una población infantil particularmente vulnerable. Además, se evaluaron de manera cuantitativa, prospectiva y sistemática un conjunto exhaustivo de variables a nivel del niño, la madre y el hogar, ofreciendo un enfoque integral para el estudio de la DCI.

Dentro de las limitaciones se destaca que algunas variables fueron autoinformadas lo que podría introducir sesgos como el de deseabilidad social. Además, el cuestionario de conocimientos si bien no fue validado, está basado en preguntas de encuestas de salud y nutrición previas como la ENDES. Otra limitación fue que no se aplicó un muestreo probabilístico para seleccionar la muestra lo cual podría limitar la extrapolación de resultados; sin embargo, el enrolamiento censal de todos los nacidos en los EESS asegura, en parte, la representatividad de los niños para inferir los hallazgos a niños atendidos en EESS del MINSA en zonas urbanas de Huancavelica y Loreto. Asimismo, la asignación no aleatoria de los participantes a diferentes grupos de exposición podría generar una distribución desigual de los factores de confusión. Además, constituye una limitación el análisis transversal realizado pues no permite explorar la causalidad de los factores para la DCI, ni evitar el sesgo de causalidad reversa que podría existir; para mitigar en parte este problema, se recopiló información detallada sobre diversos factores de confusión y se controlaron mediante ajuste en un análisis multivariado; sin embargo, cabe resaltar además que dicho análisis consideró el ajuste por covariables siguiendo únicamente criterios estadísticos, lo que pudo desestimar el impacto de variables que teóricamente pudieran tener vinculación según la literatura en otros contextos o realidades diferentes a la estudiada.

En conclusión, se identificaron diversos factores asociados a la DCI durante el primer año de vida, con variaciones según la etapa evaluada. A los tres meses, los factores de riesgo incluyeron infecciones respiratorias, la ocupación materna en actividades laborales/educativas y antecedentes de embarazos múltiples. Al sexto mes, el uso de biberón y la lengua materna distinta al castellano aumentaron el riesgo, mientras que los factores protectores fueron madres con seis o más CPN, niños con CRED incompleto y no haber recibido leche materna en la primera hora de vida. Al noveno mes, el sexo masculino, la inseguridad alimentaria y la no participación de las madres en decisiones claves se identificaron como factores de riesgo, mientras que la falta de lactancia materna en la primera hora de vida y no recibir LME hasta los seis meses mostraron un efecto protector. Finalmente, a los doce meses, los factores de riesgo incluyeron el uso de combustibles contaminantes, la lengua materna distinta al castellano, la no participación de las madres en decisiones claves y la ocupación materna en actividades laborales/académicas. Mientras que factores protectores a esta edad fueron la presencia de diarrea o anemia, así como pertenecer al programa JUNTOS. La persistencia de la DCI como problema de salud pública en contextos vulnerables subraya la necesidad de analizar en detalle las determinantes locales para diseñar intervenciones adaptadas y efectivas.
